# Valorization of Hazelnut (*Corylus avellana* L.) Skin By-Product as a Multifunctional Ingredient for Cosmetic Emulsions

**DOI:** 10.3390/antiox14101199

**Published:** 2025-10-02

**Authors:** Teresa Mencherini, Tiziana Esposito, Francesca Sansone, Daniela Eletto, Martina Pannetta, Oihana Gordobil, Anna Lisa Piccinelli, Carlo Ferniani, Giulia Auriemma, Luca Rastrelli, Rita Patrizia Aquino

**Affiliations:** 1 Department of Pharmacy, University of Salerno, Via Giovanni Paolo II, 132, 84084 Fisciano, SA, Italy; tesposito@unisa.it (T.E.); fsansone@unisa.it (F.S.); daeletto@unisa.it (D.E.); mpannetta@unisa.it (M.P.); gauriemma@unisa.it (G.A.); aquinorp@unisa.it (R.P.A.); 2 UNESCO Chair Salerno, Plantae Medicinales Mediterraneae, University of Salerno, Via Giovanni Paolo II, 132, 84084 Fisciano, SA, Italy; 3 Chemical Engineering and Environmental Department, Faculty of Engineering of Gipuzkoa, University of the Basque Country (UPV/EHU), Europa Plaza 1, 20018 Donostia, Spain; oihana.gordobil@ehu.eus; 4 National Biodiversity Future Center (NBFC), 90133 Palermo, PA, Italy; apiccinelli@unisa.it (A.L.P.); rastrelli@unisa.it (L.R.); 5 Aphros Cosmetics Srl., Via Benevento, 1, 80030 Roccarainola, NA, Italy; info@aphroscosmetics.com

**Keywords:** roasted hazelnut skin hydroalcoholic extract (RHS-H), cosmetic formulation stability, antioxidant activity, preservative and UV-filter-enhancing properties

## Abstract

Roasted hazelnut skins (RHSs), generated as by-products of industrial hazelnut processing, were extracted by pressurized liquid extraction to yield a hydroalcoholic extract (RHS-H). The extract was rich in polyphenols (308.4 ± 4.6 mg GAE/g) and proanthocyanidins (169.3 ± 10 mg CE/g) and showed strong antioxidant activity (DPPH EC_50_ = 5.08 ± 1.08 µg/mL; TEAC = 2.82 ± 0.03 mM Trolox/mg) together with antimicrobial effects against *Staphylococcus aureus* and *Staphylococcus epidermidis*. RHS-H also enhanced the UV absorbance of synthetic UV filters. When incorporated into oil-in-water (O/W) cosmetic emulsions at low concentrations (0.2–2% *w*/*w*), RHS-H did not affect physicochemical stability: formulations maintained acceptable organoleptic properties, dermocompatible pH (4.7–5.5), electrostatic stability (ζ-potential ranging from –57 to –60 mV), and rheological behavior. Functionally, RHS-H increased the antioxidant activity of emulsions (radical scavenging > 80% vs. 52% in the blank), ensured microbial protection in challenge tests, and enhanced SPF by 9.4% at 0.2% *w*/*w*, with further improvements at higher concentrations, reaching broad-spectrum photoprotection (critical wavelength > 370 nm). Overall, RHS-H represents a natural multifunctional ingredient with antioxidant, preservative, and photoprotective properties, providing a sustainable strategy to upcycle hazelnut processing waste and reduce reliance on synthetic additives in cosmetic formulations.

## 1. Introduction

Hazelnut (*Corylus avellana* L.) seed skin accounts for approximately 2.5% of the total nut weight. This brown outer layer is typically discarded during the production of peeled whole or ground roasted hazelnuts, which are widely used in the confectionery industry. While the skin constitutes a minor portion of each nut, large-scale processing generates substantial quantities of this by-product. These skins are mainly destined for landfill or, in some cases, repurposed for animal feed or energy production. However, hazelnut skin is a rich source of bioactive compounds, particularly polyphenols such as oligomeric proanthocyanidins [1,2,3], known for their strong antioxidant properties [4,5]. The polyphenolic profile of roasted hazelnut skin extracts, previously characterized by our group [1], revealed catechin, epicatechin, and galloylated derivatives as major constituents, supporting its potential as a source of biologically active metabolites. The valorization of hazelnut skin aligns with sustainability and circular economy principles by reducing waste and enabling high-value applications from agricultural residues. The recovery of bioactive metabolites from agri-food by-product also addresses the growing demand for sustainable and effective ingredients in skincare formulations. Although European legislation [6] ensures consumer safety regarding cosmetic raw materials, several ingredient classes remain controversial. These include synthetic preservatives (e.g., methylparaben, propylparaben) and organic UV filters (e.g., benzophenone-3, cinnamates, octocrylene, avobenzone), which are associated with potential health risks and environmental toxicity. Reported effects include allergic dermatitis, skin sensitization, estrogenic activity, disruption of male reproductive functions, endocrine interference, and even carcinogenicity. In addition, their persistence in the environment contributes to marine pollution, aquatic toxicity, and coral reef bleaching [7,8]. Despite these concerns, such ingredients remain essential for protecting cosmetic formulations against microbial contamination and for shielding human skin from oxidative damage caused by ultraviolet (UV) radiation and reactive oxygen species (ROS). Microbial growth in water-containing cosmetics can alter sensory characteristics, degrade functional ingredients, and cause skin irritation or inflammation. Meanwhile, overexposure to UVB (280–320 nm) and UVA (320–400 nm) radiation accelerates skin aging, dryness, and the onset of skin cancers such as melanoma. UV-induced ROS generation damages epidermal and dermal cell DNA. Although ROS have physiological roles in cell signaling and defense, their overproduction, especially due to UV exposure, disrupts redox homeostasis and triggers oxidative stress, a major contributor to photoaging [9,10]. While the skin deploys endogenous antioxidants (e.g., glutathione, glutathione peroxidase, catalase, ubiquinol), these defenses decline with age. Therefore, topical supplementation with natural antioxidants may offer protective benefits [11]. Synthetic organic filters and antioxidant agents in cosmetic formulations can absorb harmful UV radiation and mitigate oxidative damage [12,13]. However, due to rising safety and ecological concerns, regulatory bodies may impose concentration limits or full bans on these ingredients. As a result, raw material suppliers are actively seeking natural alternatives that meet both efficacy and safety expectations [14,15]. Among promising candidates are polyphenols, multifunctional natural compounds that help plants withstand abiotic stressors like UV radiation by scavenging ROS and absorbing short-wavelength light [16]. These properties make polyphenol-rich extracts from agri-food by-products particularly attractive for cosmetic applications [17,18]. Such extracts offer dual functionality: they both support skin health by countering oxidative stress, inflammation, and cellular senescence, and enhance product safety by inhibiting microbial growth during shelf life and consumer use [7,19,20].

This study investigates the potential of a roasted hazelnut skin hydroalcoholic extract (RHS-H), obtained via a green extraction process, as a sustainable multifunctional ingredient for cosmetic use. The research includes an evaluation of its antioxidant activity and antimicrobial properties, as well as the assessment of its ability to enhance the efficacy of synthetic organic UV filters. Furthermore, RHS-H was incorporated into oil-in-water (O/W) emulsions, which were analyzed for physicochemical stability, antioxidant performance, microbiological protection, and photoprotective enhancement.

## 2. Materials and Methods

### 2.1. Chemicals, Reagents, and Microorganisms

Analytical-grade ethanol, Folin–Ciocalteu reagent, sodium carbonate (Na_2_CO_3_), gallic acid, 1,1-diphenyl-2-picrylhydrazyl radical (DPPH), (+)-catechin, 2,2′-Azino-bis(3-ethylbenzothiazoline-6-sulfonic acid) (ABTS), potassium persulfate, PBS, Propylene Glycol, vanillin, benzyl mercaptan and Trolox were purchased from Merck KGaA (Darmstadt, Germany). Glycerin, Xanthan Gum, Disodium Ethylenediaminetetraacetic acid (EDTA), Butirrospermum Parkii, Cetearyl Alcohol, Ethylhexyl Stearate, Persea Gratissima Oil, Dimethicone, Caprylic/Capric Triglyceride, Dicapryl Ether, C12-15 Alkyl Benzoate, Ethylhexyl Methoxycinnamate, Butyl Methoxydibenzoylmethane, Octocrylene, Butyl Hydroxytoluene were acquired from A.C.E.F. S.p.A. (Fiorenzuola d’Arda, PC, Italy). Phenoxyethanol (and) Ethylhexylglycerin was purchased from Thor Especialidades, S.A. (Castellgalí, Barcelona, Spain), and Glyceryl Stearate (and) Peg-100 Stearate from BASF (Ludwigshafen, Germany).

### 2.2. Hazelnut Skin Biomass and Extraction Procedure

Roasted hazelnut skins (RHS), obtained as industrial by-products from a processing company in Avellino (Campania, Italy), were milled and sieved to obtain a particle size fraction of 300–600 µm. The extract (RHS-H) was prepared using pressurized liquid extraction (PLE) with aqueous ethanol (30%, *v*/*v*) under previously described conditions [21]. Briefly, the extraction was carried out with five cycles at 125 °C and 1500 psi, and the solvent was subsequently removed at 40 °C under reduced pressure by a rotary evaporator (Heidolph Hei-VAP Value Digital, Schwabach, Germany), followed by lyophilization by a freeze dryer Alpha 1–2 LD (Christ, Osterode am Harz, Germany). The extraction yield was determined gravimetrically and was 33.2 g per 100 g of dry material.

### 2.3. Hazelnut Skin Hydroalcoholic Extract (RHS-H) Characterization

#### 2.3.1. Total Phenolic Content

The polyphenolic content of RHS-H was quantified using the Folin–Ciocalteu colorimetric assay as reported by Piccinelli et al. 2016 [1] with some modifications. Gallic acid served as a calibration standard. Gallic acid served as a calibration standard curve (y = 0.0207x − 0.4233, R^2^ = 0.9968, concentration range 20–75 μg /mL), and results were expressed as mg of gallic acid equivalents (GAE) per gram of extract.

50 µL of a 1:10 dilution of RHS-H (100 µg/mL) with H_2_O was added to 450 µL of Folin’s reagent. After 3 min, 500 µL of a 10% solution of sodium carbonate (Na_2_CO_3_) were added. The samples were incubated in the dark for 1 h and the absorbances were evaluated with a spectrophotometer (Analytic Jena Specord 200 plus, Konrad-Zuse-Straße, Leer, Germany) at λ 723 nm.

#### 2.3.2. Determination of Total Proanthocyanidin (Pas) Content (Vanillic Assay)

For determination of PAs content, the vanillin assay was employed as reported by Butler et al. 1982 [22] with some modifications. In brief, 40 μL of RHS-H (200 μg/mL in acetic acid) were mixed with 200 μL of vanillin solution (0.5% vanillin reagent, *w*/*v*, in acetic acid, plus 4% of concentrated HCl) in a 96-well microplate. After 5 min at room temperature, absorbance was read at 510 nm using a microplate spectrophotometer reader Multiskan Go (ThermoFisher Scientific, Milan, Italy). A control without vanillin reagent and a blank with acetic acid instead of the sample were included in the assay. The concentration of total PAs was estimated from a calibration curve using catechin (range 1–100 μg/mL, prepared in acetic acid) and the data were expressed as catechin equivalent (CE mg/ g extract, means ± S.D. of three determinations).

#### 2.3.3. Thiolysis

An acid-catalyzed depolymerisation in the presence of benzyl mercaptan as nucleophilic reagent (thiolysis), coupled to HPLC-UV-HRMS analysis, was applied to determine the nature and the proportion of the flavanol units and average Degree of Polymerization (mDP) of PA mixtures [23]. The thiolysis reaction was carried out following the method described by Piccinelli et al. 2016 [1], with no substantial modifications. Briefly, the reaction was initiated by adding 50 μL acidified methanol (HCl, 3.3%, *v*/*v*) and 100 μL of benzyl mercaptan solution (5%, *v*/*v*, in methanol) to 50 μL of the extract (4 mg/mL in methanol). After 30 min at 40 °C, 200 μL of water were added to the reaction mixture and the samples were analyzed by HPLC-UV analysis using the same conditions reported in Piccinelli et al. 2016 [1]. Experiments were performed in triplicate.

#### 2.3.4. Bleaching of the Radical 1,1-Diphenyl-2-picrylhydrazyl (DPPH Test)

The antioxidant potential was evaluated through the reduction of the stable DPPH radical, according to Esposito et al. 2019 [24] with slight modifications. The effective concentration at 50% inhibition (EC_50_) was determined, corresponding to the sample concentration required to quench half of the radical population. The concentration of DPPH in the reaction medium was calculated from a calibration curve (y = 0.0211x + 0.0380, R^2^ = 0.9975, range = 5–36 µg/mL). To 1.5 mL of a methanolic solution of DPPH (3.6 mg/100 mL) prepared daily, 37.5 μL of RHS-H (from 6.25 to 50 μg/mL) or positive control (catechin, from 5 to 20 μg/mL) were added. All tests were performed in triplicate.

#### 2.3.5. Trolox Equivalent Antioxidant Capacity (TEAC) Assay

The antioxidant activity of RHS-H was further evaluated by the ABTS·^+^ radical cation decolorization method, following Esposito et al. 2019 [24] with minor modifications. The ABTS·^+^ radical was generated by mixing 7.0 mM ABTS with 2.45 mM potassium persulfate (1:1, *v*/*v*) and allowing the solution to react overnight in the dark at room temperature. The resulting radical stock was diluted with PBS (pH 7.4) to obtain a working solution with an absorbance of 0.70 ± 0.05 at 734 nm. For the assay, 15 µL of RHS-H (0.005125–0.001 mg/mL) or catechin standard (0.003–0.0075 mM) were added to 1485 µL of the ABTS·^+^ working solution. After 1 min of incubation at room temperature, absorbance was recorded at 734 nm using a Thermo Evolution 201 UV–vis spectrophotometer (Thermo Fisher Scientific, Milan, Italy). PBS without ABTS·^+^ served as the blank. Antioxidant capacity was expressed as Trolox Equivalent Antioxidant Capacity (TEAC, mmol Trolox/mg extract or mmol compound), calculated from a Trolox calibration curve.

#### 2.3.6. UV Spectra and In Vitro Sun Protection Efficacy

UV spectra of an aqueous solution with 0.1% propylene glycol of 100 µg/mL of RHS-H alone or mixed with the synthetic UV filter Ethylhexyl Methoxycinnamate, Butyl Methoxydibenzoylmethane, or Octocrylene (5–10 µg/mL dissolved in ethanol) were acquired (UV/Vis spectrometer Specord 200 plus Sotax powered by AnalitykJena, Jena, Germany) in the range 220 and 400 nm. Spectra were recorded against solvent-matched blanks: water with 0.1% propylene glycol for RHS-H; ethanol for individual UV filters; and the corresponding water/propylene glycol–ethanol mixture (same proportions as in the test samples) for RHS-H + filter mixtures. In vitro Sun Protection Factor (SPF) was determined spectrophotometrically between 290 and 320 nm (in 5 nm increments), applying the Mansur equation [25]:

(1) $$\mathrm{SPF}\;=\;\mathrm{CF}\;\times \sum_{290}^{320}\mathrm{EE}\;\left(\lambda \right)\times I\;\left(\lambda \right)\times \mathrm{Abs}\;\left(\lambda \right)$$

where EE (λ) = Erythemal Effect spectrum; I (λ) = solar intensity spectrum; Abs (λ) = absorbance of the sample; CF = Correction Factor (= 10).

The constants of EE × I were obtained by Sayre et al. 1979 [26] and reported in Table 1.

#### 2.3.7. Determination of Minimum Bactericidal Concentration (MBC)

The antibacterial activity of RHS-H was evaluated against *S. aureus* (ATCC 6538), *S. epidermidis* (ATCC 03111), *Escherichia coli* (ATCC 25922), and *Pseudomonas aeruginosa* (ATCC 9027). Microorganisms were obtained from LGC Standards S.r.L. (Milan, Italy). To determine the in vitro Minimum Bactericidal Concentration (MBC) of the extract, micro-broth dilution assays were performed in line with the Clinical and Laboratory Standards Institute (CLSI) guidelines. In detail, two colonies of each bacterial strain from Mueller–Hinton Agar (MHA, Thermo Fisher Scientific, Milan, Italy) were initially resuspended and grown overnight in Mueller–Hinton Broth (MHB, Thermo Fisher Scientific, Milan, Italy) at 37 °C. Inocula were then prepared by diluting overnight cultures at a concentration of ≈ 10^6^ CFU/mL in fresh MHB. From this suspension, 100 μL was used to inoculate flat-bottom 96-well polystyrene microtiter plates containing two-fold serial dilutions of the extract from 1 mg/mL to 0.25 mg/mL. The minimum bactericidal concentration (MBC) was determined by plating 100 μL of each well or proper serial dilutions on MHA incubated at 37 °C for 24 h. The MBC was identified as the lowest concentration that prevents any microbial growth on an agar plate. Each assay was performed in triplicate on separate days.

### 2.4. Development and Production of O/W Cosmetic Emulsions

Four cosmetic Oil-in-Water (O/W) emulsions were developed (Table 2). F1, containing synthetic UV filters and preservatives, was used as the control (blank) formulation. F2 was prepared to evaluate RHS-H as a preservative, while F3 combined RHS-H with a reduced amount of synthetic preservative (co-preservative system). To assess the contribution of RHS-H to UV protection, F4 (RHS-H alone) and F3 (RHS-H with UV filters) were compared. For the in vitro SPF evaluation, additional emulsions F5–F8 were prepared: F5 and F6 were identical to F3 but contained 1% and 2% RHS-H, respectively, whereas F7 and F8 were identical to F4 but with 1% and 2% RHS-H.

For preparation, the aqueous phase (water + EDTA) was heated to 70 ± 2 °C, and Xanthan Gum pre-dispersed in Glycerin was added. The oily phase was separately heated to the same temperature, and UV filters (Ethylhexyl Methoxycinnamate, Butyl Methoxydibenzoylmethane, and Octocrylene) solubilized in C12-15 Alkyl Benzoate were incorporated just before emulsification. The oil phase was then slowly added to the aqueous phase and emulsified with a turboemulsifier (10 min, 8000 rpm), followed by manual mixing until cooling. At 33 ± 2 °C, preservatives (Phase C) were added to F1, F2, and F3. RHS-H, pre-dispersed in Glycerin and water (Phase D), was incorporated at 25 ± 2 °C in F2, F3, and F4.

### 2.5. Physico-Chemical Stability Tests

#### 2.5.1. Stability Tests at 25 °C Up to 6 Months

The O/W emulsions were stored at 25 ± 1.0 °C for 6 months. At established times (48 h; 15 and 180 days), the stability in the centrifuge and pH and rheological behavior values were verified.

#### 2.5.2. Heat Shock Cycles

The O/W emulsions were subjected to 3 cycles of heating and cooling. The formulations were stored at 4 °C for 24 h, removed, and directly placed at 50 °C for another 24 h. After 3 cycles, the physicochemical parameters (centrifuge stability, pH, and rheological behavior) of the emulsions were checked.

#### 2.5.3. Centrifugation Stability Test

For each O/W emulsion, 7 g of product were transferred into centrifuge tubes and subjected to accelerated stress conditions using a Labofuge 200 Centrifuge (Thermo Scientific, Waltham, MA, USA). Samples were first spun at 4000 rpm for 30 min. If no evidence of phase separation (e.g., creaming, flocculation, or coalescence) was detected, the same aliquot was further centrifuged at 5300 rpm for 15 min to confirm stability under more stringent conditions [27].

#### 2.5.4. Organoleptic and pH Determination

Macroscopic inspection was carried out to assess visual and olfactory characteristics of the emulsions, including homogeneity, color, appearance, and odor [28]. pH was measured at 25 ± 0.3 °C using a Seven Easy digital pH meter (Mettler Toledo, OH, USA) calibrated with standard buffer solutions (pH 4.04, 7.00, and 9.21). Each value represents the mean of three independent measurements [27].

#### 2.5.5. Zeta Potential

Electrophoretic light scattering (ELS) was employed to determine zeta potential, using a Litesizer 500 Particle Analyzer (Anton Paar GmbH, Graz, Austria) equipped with a 658 nm diode laser, delivering 40 mW, and temperature-controlled at 25 °C. The Smoluchowski approximation was applied to convert electrophoretic mobility into zeta potential values, a method commonly used for particles suspended in aqueous solutions with moderate electrolyte concentrations. Kalliope software version 1.8 was used for data acquisition and analysis. Zeta potential measurements were conducted by diluting the samples at a 1:500 (*v*/*v*) ratio, followed by 30 min of stirring. The analysis was performed in a polycarbonate omega cuvette cell with graphite electrodes.

### 2.6. Rheological Behavior Characterization

Rheological behavior of F1 and F3 emulsions was analyzed with an MCR102 rheometer (Anton Paar, Graz, Austria) equipped with a CP50-1 cone–plate system (diameter 49.980 mm, cone angle 0.989°, truncation 99 µm). Data acquisition and processing were performed using RheoCompass™ software [27]. Both steady-shear and oscillatory tests were conducted. Flow properties were determined by recording viscosity (mPa·s) across shear rates ranging from 0.01 to 1000 s^−1^ (21 points per decade) within 30 s. Amplitude sweep experiments, performed at a constant frequency of 10 s^−1^ with strain increasing from 0.01% to 100% (logarithmic mode), were used to define the Linear Viscoelastic Region (LVR). Frequency sweep measurements were then carried out at a constant stress within the LVR, over an angular frequency range of 0.1–100 rad·s^−1^. All experiments were performed in triplicate at 25.0 ± 1.0 °C.

### 2.7. Evaluation of Free Radical Scavenging Activity of Formulations

To evaluate the DPPH radical scavenger ability of the cosmetic emulsions (F1-F4), 10 mL of methanol was added to 1 g of each cream, separately. The suspension was vortexed for 30 s, subjected to ultrasound (40 KHz) in a water bath for 20 min, and centrifuged at 5300 rpm for 15 min. 2.5 mL of supernatant was added to 2.5 mL of a methanolic solution of DPPH. Samples were kept in the dark at room temperature for 30 min, and the decrease in absorbance at 517 nm was recorded after 10 and 30 min (spectrophotometer Analytic Jena Specord 200 plus, Thermo Fischer Scientific, Leer, Germany). The results were expressed as Radical Scavenging Activity percentage (RSA%):

$$\mathrm{RSA}\%=\;\frac{Ac-As\;}{Ac}\times 100$$

where *Ac* is the absorbance of the control (without the sample) and *As* is the absorbance of the sample. All tests were performed in triplicate.

### 2.8. Preservative Efficacy Testing (Challenge Test)

An in vitro challenge test, carried out in accordance with UNI EN ISO 11930:2022 [29], was used to evaluate the preservative efficacy of the formulations.

Emulsions were inoculated with reference bacterial and fungal strains, and microbial counts were monitored over time to verify compliance with acceptance criteria. Each formulation was challenged, respectively, with *Staphylococcus aureus* (ATCC 6538), *Pseudomonas aeruginosa* (ATCC 9027), *Candida albicans* (ATCC 10231), and *Aspergillus brasiliensis* (ATCC 16404). Microorganisms were obtained from LGC Standards S.r.L. (Milan, Italy).

Formulations were divided into 10 mg aliquots and inoculated with bacterial suspensions at 10^6^/mL or fungal suspensions at 10^5^/mL. Samples were incubated at 20–25 °C. At different time points, 2, 7, 14, and 28 days, one mg aliquots were serially diluted in Muller-Hinton broth or in Yeast nitrogen base broth (YNB, Thermo Fisher Scientific, Milan, Italy) and plated in duplicate on Muller-Hinton Agar (for bacteria) or Sabouraud dextrose agar (SDA, Thermo Fisher Scientific, Milan, Italy) (for fungi). Plates were incubated at 30–35 °C for up to 3 days for bacteria and 20–25 °C for up to 5 days for fungi. Raw microbial counts were converted to log_10_ values for analysis.

### 2.9. In Vitro Sun Protection Factor (SPF) Evaluation of the O/W Emulsions

Sun protection factor (SPF) of O/W emulsion systems was estimated through spectrophotometric measurements on film-coated substrates, following the in vitro Diffey–Robson approach [30]. This method provides a more realistic estimation of SPF than solution-based tests. The used method considers the full UV spectrum (290–400 nm), allowing for a rapid assessment of the SPF as well as the study of the product against long-wavelength ultraviolet radiation. Emulsions were applied at 2 mg/cm^2^ to quartz slides and dried for a few minutes before UV transmittance measurements. A quartz slide was used as a reference. Due to the heterogeneity of the samples during the application of the emulsion on the slides, 20–30 replicas were analyzed. UV transmittance was measured in a UV-vis-NIR Shimadzu 3600 spectrophotometer from 290 to 400 nm at 0.5 nm intervals. The collected transmittance spectra were used to calculate in vitro Sun Protection Factor (SPF) (Equation (2)).

(2) $$\mathrm{SPF}=\frac{\sum_{290}^{400}E\lambda \;B\lambda }{\sum_{290}^{400}\frac{E\lambda \;B\lambda }{MPF\lambda }}$$

where *Eλ* = CIE erythemal spectral effectiveness, *Bλ* = solar spectral irradiance, and *Fλ* = spectral transmittance of the solid substrate/spectral transmittance of the sample.

In addition, the UVA/UVB ratio and the critical wavelength (Cλ) were calculated from the absorbance spectrum using Equations (3) and (4), respectively.

(3) $$\frac{UVA}{UVB\;}\;\mathrm{ratio}=\frac{\frac{\sum_{320}^{400}A\lambda \;\Delta \lambda }{\sum_{320}^{400}\Delta \lambda }}{\frac{\sum_{280}^{320}A\lambda \;\Delta \lambda }{\sum_{280}^{320}\Delta \lambda }}$$

(4) $$C\lambda =\int_{280}^{C\lambda }A\lambda \;d\lambda =0.9\;\int_{280}^{400}A\lambda \;d\lambda$$

### 2.10. Statistical Analyses

Statistical analyses were performed using GraphPad Prism version 10.0 (GraphPad Software, San Diego, CA, USA). One-way analysis of variance (ANOVA) followed by Tukey’s post hoc test was used to assess significant differences among the obtained results. Statistical significance was set at *p* < 0.05.

## 3. Results and Discussion

### 3.1. Production of RHS-H and Chemical Characterization

Hazelnut skins, a by-product of industrial hazelnut processing, were extracted using pressurized liquid extraction (PLE) with aqueous ethanol, a green and efficient technique previously optimized by our group [1]. The process provided high yields (33.2 ± 0.7 g/100 g dry skin) and ensured food-grade compatibility, allowing the production of RHS-H suitable for cosmetic applications. The resulting extract (RHS-H) exhibited a total polyphenol content of 308.4 ± 4.6 mg GAE/g extract and a high concentration of proanthocyanidins (PAs) (169.3 ± 10 mg CE/g extract), comparable to that of one of the most recognized commercial PA-rich extracts, Pycnogenol^®^ (126.1 ± 2.0 mg CE/g extract), derived from the bark of the French maritime pine Pinus pinaster. PAs of RHS-H showed an average degree of polymerization (mDP) of 8.6 ± 0.6. The flavanol profile of RHS-H, characterized by catechin, epicatechin, and galloylated derivatives such as catechin-3-O-gallate, epigallocatechin, gallocatechin, and gallocatechin-3-O-gallate, was consistent with the composition previously identified in detail by our group [1].

### 3.2. RHS-H Functional Characterization

#### 3.2.1. Scavenging Activity Against DPPH and ABTS Radicals

Identifying natural compounds with antioxidant activity is critical for mitigating damage caused by reactive oxygen species (ROS) to human cellular structures. Among the most common exogenous sources of ROS are air pollution, solar radiation, and cigarette smoke, which promote the accumulation of free radicals in the stratum corneum. These unstable molecules can damage membrane lipids, proteins, and DNA, disrupt collagen fibers organization, and contribute to premature skin aging and the development of skin disorders. The topical application of polyphenol-rich extracts may help counteract these effects by neutralizing free radicals or converting them into less reactive species [31].

RHS-H exhibited strong radical scavenging activity, with an EC_50_ value of 5.08 ± 1.08 µg/mL against DPPH radicals, outperforming pure catechin (EC_50_ = 15.82 ± 1.27 µg/mL). In the ABTS assay, RHS-H showed a Trolox Equivalent Antioxidant Capacity (TEAC) of 2.82 ± 0.03 mM Trolox/mg extract, compared to 3.27 ± 0.01 mM Trolox/mg for catechin. The antioxidant potential of roasted hazelnut skin is well-documented and is primarily attributed to the presence of condensed tannins, such as proanthocyanidins, whose flavanol subunits can donate electrons or hydrogen atoms to neutralize radical species and prevent propagation reactions [20]. Based on these properties, some authors have proposed the use of hazelnut skin extracts, obtained using either conventional solvents or innovative systems such as Natural Deep Eutectic Solvents (NADES, a class of bio-based, low-toxicity, and biodegradable green solvents with high solubilizing power), for incorporation into cosmetic formulations [32].

#### 3.2.2. UV Absorbance and In Vitro SPF of Ingredients: RHS-H and Synthetic UV Filters

Beyond the use of exogenous antioxidants, protecting the skin from premature aging and UV-induced damage also requires the use of skincare products containing chemical UV filters that absorb harmful ultraviolet radiation. Polyphenol-rich extracts with antioxidant properties may enhance the performance of synthetic UV filters, allowing for lower active concentrations.

**Figure 1:**
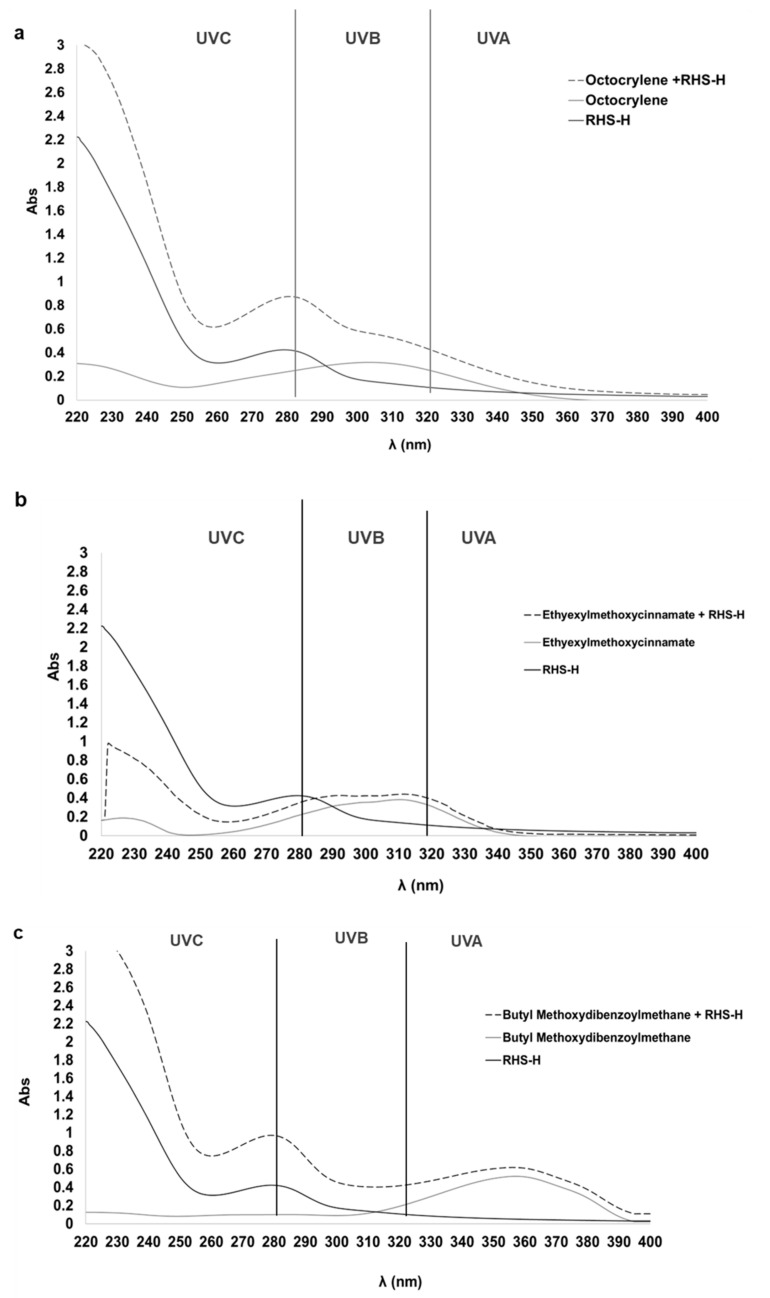
UV absorption spectra (220–400 nm) of RHS-H, synthetic UV filters, and their combinations. (**a**) Octocrylene (light gray line), RHS-H (dark gray line), and their mixture (dotted line); (**b**) Ethylhexyl Methoxycinnamate (light gray), RHS-H (dark gray), and their mixture (dotted); (**c**) Butyl Methoxydibenzoylmethane (light gray), RHS-H (dark gray), and their mixture (dotted). RHS-H enhances UV absorbance, particularly in the UVC and UVB regions.

RHS-H displayed effective UV absorption, particularly in the UVC (<280 nm) and UVB (280–320 nm) ranges. The extract showed a peak at 280 nm (Figure 1a–c, dark gray line), characteristic of proanthocyanidins [33] and exhibited strong absorption in the UVC region. When combined with synthetic filters, RHS-H enhanced the overall UV absorption, especially in the UVC and UVB ranges. For Octocrylene, which showed a typical absorption peak between 280 and 320 nm (Figure 1a, light gray line), the addition of RHS-H (dotted line) resulted in a 3.58-fold increase in absorbance. Ethylhexyl Methoxycinnamate combined with RHS-H (Figure 1b, dotted line) exhibited a 1.14-fold increase in absorbance at 310 nm compared to the pure filter. For Butyl Methoxydibenzoylmethane, the addition of RHS-H (Figure 1c, dotted line) broadened the absorption spectrum and enhanced absorption by 9.57-fold at 290 nm and 1.18-fold across the 320–400 nm UVA range.

These results indicate that RHS-H can significantly boost the UV-absorbing capacity of synthetic filters. Compared to the study by Ivanović et al. 2020 [34], which found negligible improvement when combining benzophenone-3 with hazelnut skin extracts (10 mg/L, aqueous ethanol 10–50–96%, *v*/*v*), our new findings demonstrated markedly superior performance for RHS-H. The synergistic interaction between antioxidant extracts from agri-food by-product and UV filters has also been reported in other studies. Galanakis et al. 2018 [35,36] showed that polyphenols recovered from olive mill wastewater enhanced the UV absorption of both chemical and physical filters, outperforming vitamins C and E, with the booster effect being proportional to polyphenol concentration (0–15 mg/L).

The protective capacity of a formulation against UV radiation is expressed as its Sun Protection Factor (SPF) value. SPF is calculated as the ratio between the minimum UV dose required to produce erythema on protected skin and that on unprotected skin [37]. While in vivo testing is required for regulatory purposes, in vitro spectrophotometric methods are widely used during formulation development. In this study, SPF values were calculated using the Mansur equation, which, despite being limited to estimating protection based on absorbance in the UVB range (290–320 nm) and assuming a linear correlation between absorbance and protective efficacy, provides a rapid and practical screening tool for evaluating substances in solution. RHS-H exhibited an in vitro SPF of 3.63 at 0.1 mg/mL and significantly improved the SPF of the synthetic UV filters. When blended with RHS-H, the SPF values of Octocrylene, Ethylhexyl Methoxycinnamate, and Butyl Methoxydibenzoylmethane increased from 4.90, 3.88, and 3.98 to 9.13, 9.73, and 7.33, respectively (Table 3).

These findings are in line with other studies reporting high SPF values for extracts rich in flavan-3-ols and B-type procyanidins. For instance, Liu et al. 2024 [38] reported that Cinnamomum camphora extracts exhibited higher UVB-blocking activity than synthetic filters like benzophenone and homosalate. Similarly, Ferreira et al. 2023 [39] identified polyphenol-rich extracts from onion peel and passion fruit as natural UV filters, with SPF values correlating with gallic acid, resveratrol, and flavonoid content. Extracts from Washingtonia filifera seeds demonstrated SPF values up to 3.35 at 100 µg/mL [40].

The UV-shielding capacity of flavonoids is attributed to their aromatic rings and π-conjugated electronic systems, which enable efficient absorption of UV radiation energy [13].

#### 3.2.3. Antimicrobial Activity

The antimicrobial activity of RHS-H was assessed by determining the Minimum Bactericidal Concentration (MBC) against Gram-positive bacteria (*S. aureus* and *S. epidermidis*) and Gram-negative strains (*Escherichia coli* and *Pseudomonas aeruginosa*). *S. aureus* is a major pathogen responsible for superficial and deep skin infections, due to its immune evasion mechanisms and production of multiple virulence factors promoting colonization and inflammation [41]. *S. epidermidis*, although commonly regarded as a skin commensal, has emerged as an opportunistic pathogen, especially in immunocompromised individuals and in biofilm-associated infections, due to its antimicrobial resistance and immunomodulatory properties [42].

**Figure 2:**
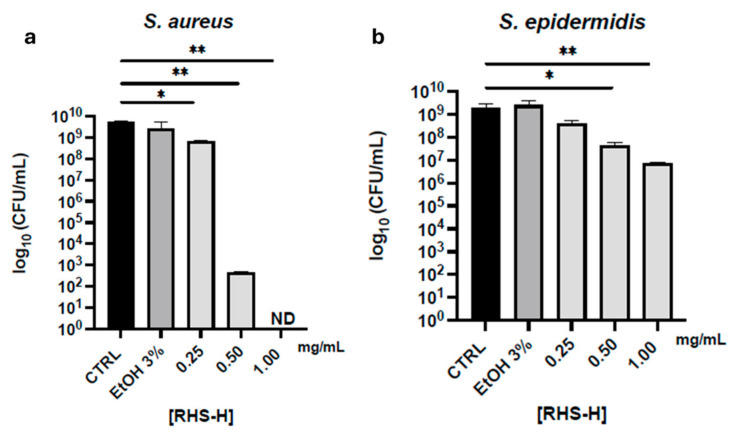
Bactericidal activity of RHS-H extract. (**a**) *S. aureus* and (**b**) *S. epidermidis* cultures were incubated with increasing concentrations of RHS-H for 24 h at 37 °C. Colony-forming units (CFU/mL) were quantified to assess viability. CTRL: untreated control; EtOH 3%: solvent control. Data are expressed as mean ± SD of three independent experiments. * *p* < 0.05, ** *p* < 0.01 (one-way ANOVA with Tukey’s post hoc test).

As shown in Figure 2a, RHS-H at 1 mg/mL achieved complete growth inhibition of *S. aureus* (consistent with MBC criteria), while a concentration of 0.5 mg/mL reduced bacterial viability by over 70%. No significant bactericidal effect was observed at lower concentrations. In the case of *S. epidermidis*, only a limited reduction (~20%) in viability was recorded at the highest concentration tested, with no complete bactericidal activity. No inhibitory effect was observed against *E. coli* and *P. aeruginosa* at concentrations up to 1 mg/mL.

The selective antibacterial activity of RHS-H against *S. aureus*, and to a lesser extent *S. epidermidis*, is consistent with previous findings on polyphenol-rich hazelnut by-product extracts. Di Michele et al. 2021 [43] reported that ethanol-based extracts from hazelnut shells exhibited higher efficacy against Gram-positive strains, while Gram-negative bacteria remained unaffected. Similarly, Esposito et al. 2020 [21] demonstrated strong antibacterial activity of hazelnut skin extract, incorporated into biodegradable films, specifically against *S. aureus*. These observations support the hypothesis that proanthocyanidins and flavanols in RHS-H exert membrane-disrupting effects in Gram-positive bacteria. In contrast, the outer lipopolysaccharide layer of Gram-negative species likely limits polyphenol penetration, interaction with the peptidoglycan layer, and overall activity [39,44].

### 3.3. Design and Development of O/W Cosmetic Emulsions

Based on the promising functional results of RHS-H, a series of Oil-in-Water (O/W) cosmetic emulsions was developed. A base formulation (F1) was designed as a control, while RHS-H was incorporated into formulations F2–F4 to evaluate its performance as a preservative, antioxidant, and UV filter booster.

O/W emulsions were selected as the most suitable delivery system for RHS-H due to the extract composition of molecules with varying polarity. To ensure high skin compatibility and maximize multifunctional efficacy, all formulations were prepared using ingredients approved under European Cosmetic Regulation No. 1223/2009 [6]. The emulsions were stabilized with a non-ionic emulsifier system and featured a lipid phase composed of natural emollients, including Butyrospermum parkii (shea butter), Caprylic/Capric Triglyceride (derived from coconut oil), and *Persea gratissima* (avocado) oil, all known for their moisturizing and skin-regenerating properties. Dimethicone was added to improve spreadability and minimize the whitening effect commonly observed in O/W formulations. Additional emollient and humectant agents included Ethylhexyl Stearate, Dicaprylyl Ether, and Glycerin [27]. Formulations F1, F3, and F4 contained a preservative blend of Phenoxyethanol and Ethylhexylglycerin. The latter also acts as a skin conditioning agent and synergistically enhances the antimicrobial efficacy of Phenoxyethanol, offering broad-spectrum protection against bacteria, yeasts, and molds. Photoprotection in F1–F3 was achieved by combining the UVB filters Octocrylene and Ethylhexyl Methoxycinnamate with the UVA filter Butyl Methoxydibenzoylmethane, which are commonly used in cosmetic sunscreen formulations [45]. The complete qualitative and quantitative composition of the emulsions is reported in Table 2.

### 3.4. Stability Studies of O/W Emulsions F1–F4

Following the development of formulations F1–F4, stability tests were performed to evaluate their organoleptic and physicochemical properties, including visual appearance, physical stability, pH, zeta potential, and rheological behavior. Assessments were conducted at predefined intervals during storage at 25 °C and after three thermal shock cycles (alternating between 4 °C and 50 °C).

**Figure 3:**
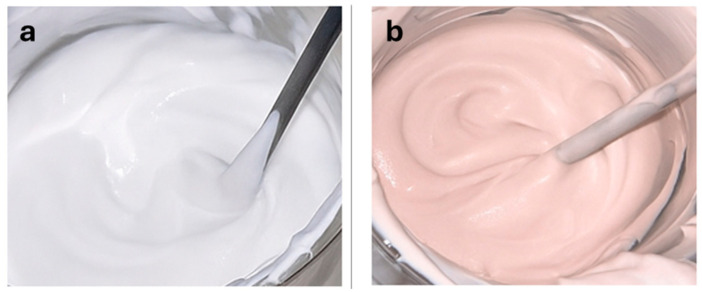
Macroscopic appearance of O/W emulsions: (**a**) control formulation F1 (white); (**b**) formulations F2–F4 containing RHS-H (pinkish).

Organoleptic evaluation is crucial to ensure that sensory properties, such as appearance, color, and odor, remain acceptable over time. This step also verifies that the inclusion of the extract does not compromise consumer appeal. All formulations (F1–F4) retained a homogeneous appearance, with RHS-H visibly well dispersed. As shown in Figure 3a,b, incorporation of RHS-H (0.2% *w*/*w*), due to the natural brownie color of the extract, changed the color of the base emulsion from white (F1) to a uniform pinkish hue in F2–F4, without sedimentation or phase separation during the entire observation period.

Centrifugation testing, which applies mechanical stress to accelerate potential destabilization, confirmed physical stability in all formulations, with no evidence of creaming, precipitation, or phase separation.

**Figure 4:**
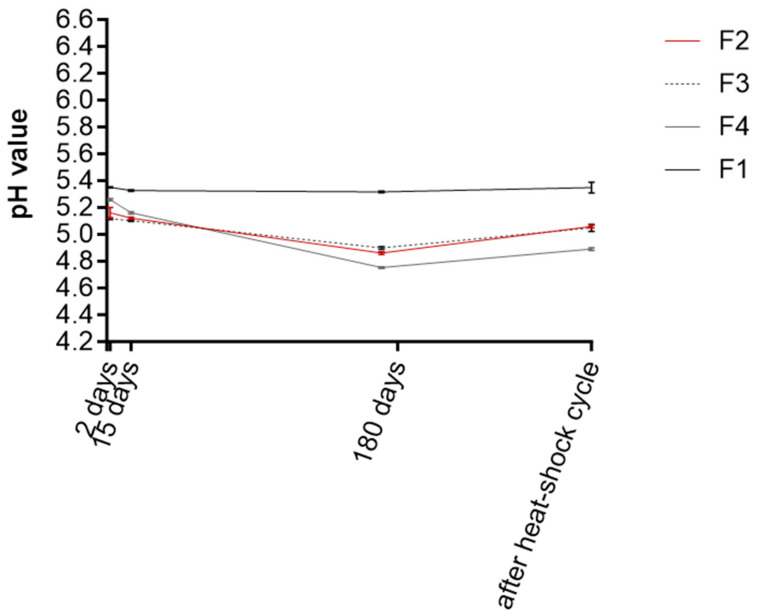
pH values of emulsions F1–F4 measured at 48 h, 15 days, 180 days, and after thermal shock cycles. Data are presented as mean ± SD (n = 3).

pH monitoring is a critical parameter, as deviations may indicate microbial contamination or instability. Furthermore, pH must remain within the skin-compatible range to avoid irritation. Ideal values for semisolid formulations intended for facial use range between 4.0 and 6.0 [46]. Elevated pH may promote microbial growth, whereas highly acidic pH could disrupt the natural skin microbiota. Moreover, many common cosmetic preservatives, particularly organic acids, show higher efficacy in acidic environments due to the predominance of their undissociated active form [47]. Throughout the test period, the pH of F1 remained stable between 5.31 and 5.35 (Figure 4). In contrast, formulations containing RHS-H (F2–F4) showed slightly lower pH values at baseline (4.90–5.27) and further reduction after 180 days (4.75–4.90), although still within the acceptable dermocosmetic range (4.70–5.50). This pH decrease is attributed to the mildly acidic polyphenols in RHS-H, consistent with previous findings. For instance, Pinto et al. 2021 [46] reported a similar pH drop in an O/W emulsion containing *Castanea sativa* shell extract. Ferreira et al. 2023 [39] also observed that polyphenol-rich extracts from onion and passion fruit peel reduce pH due to the acidic nature of phenolic compounds.

**Figure 5:**
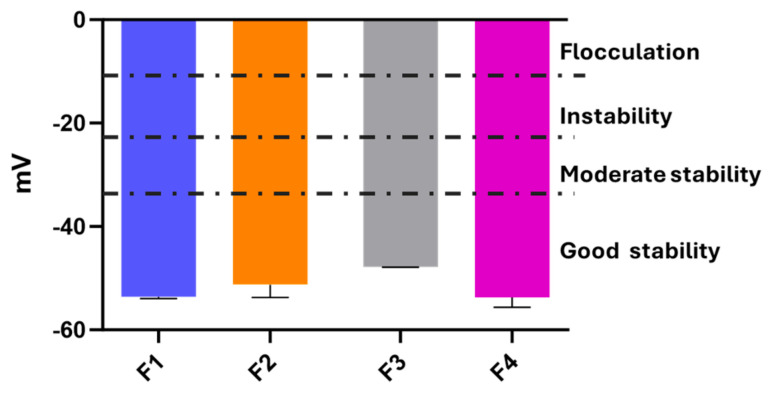
Zeta potential values of emulsions F1–F4 after 180 days and heat-shock treatment.

Zeta potential, an indicator of surface charge and emulsion stability, was also measured. Values exceeding ±30 mV are generally considered indicative of good physical stability, as electrostatic repulsion prevents droplet coalescence [48]. After six months and shock stress, all formulations exhibited zeta potential values ranging from –57 to –60 mV (Figure 5), confirming robust colloidal stability. Despite the use of a non-ionic emulsifier, negative surface charges were expected due to the inclusion of xanthan gum, whose glucuronic acid residues contribute to the overall negative charge [49,50,51].

### 3.5. Rheological Behavior Analysis of O/W Emulsions

Rheological properties play a critical role in cosmetic emulsions, influencing not only product stability but also consumer perception. Comprehensive rheological evaluation is essential to assess formulation robustness and monitor changes due to active ingredients and storage conditions [52].

#### 3.5.1. Flow Curve Analysis

**Figure 6:**
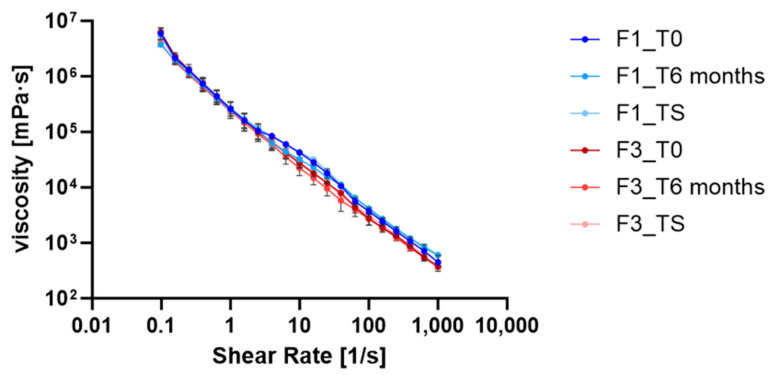
Flow curves obtained from viscosity (η) as a function of shear rate (γ), for the different formulations (F1 and F3) at different storage conditions. Rheological data are shown as mean ± SD (n = 3).

Flow curve analysis was performed on F1 (blank) and F3 (containing RHS-H) emulsions to evaluate the impact of the extract on rheological behavior and storage stability. Measurements were taken 24 h after preparation (T0), after six months at 25 °C (T6), and after thermal shock cycles (TS) (Figure 6).

Both emulsions exhibited shear-thinning (pseudoplastic) behavior, with dynamic viscosity (η) decreasing as shear rate increased. F1 showed viscosity values ranging from 5.06 × 10^6^ to 552.52 mPa·s, while F3 ranged from 5.10 × 10^6^ to 580.88 mPa·s.

This behavior is typical of non-Newtonian fluids such as creams, gels, lotions, and emulsions, and it suggests a semi-flexible molecular structure where aggregates quickly break down and droplets align under high shear, facilitating ease of spreading on the skin [53]. The non-Newtonian behavior observed is largely due to the asymmetric particles within the emulsions, which is a critical attribute for cosmetic products that undergo deformation during application [46,54]. The presence of RHS-H did not affect the flow profile (Figure 6), consistent with previous findings for emulsions containing *Castanea sativa* bur extracts (Esposito et al., 2021) [27]. The persistence of this shear-thinning profile after six months and thermal stress confirms the rheological stability of both formulations [39]. This is critical for ensuring uniform spreadability and product integrity over time [46,54,55].

#### 3.5.2. Amplitude Sweep Test

Oscillatory amplitude sweep tests were conducted to assess the rigidity and internal structure of the emulsions. The storage modulus (G′) reflects elastic behavior, while the loss modulus (G″) reflects viscous response [56]. The test was carried out at a constant frequency of 10 rad/s, with strain (γ) ranging from 0.01% to 100% at 25 °C.

**Figure 7:**
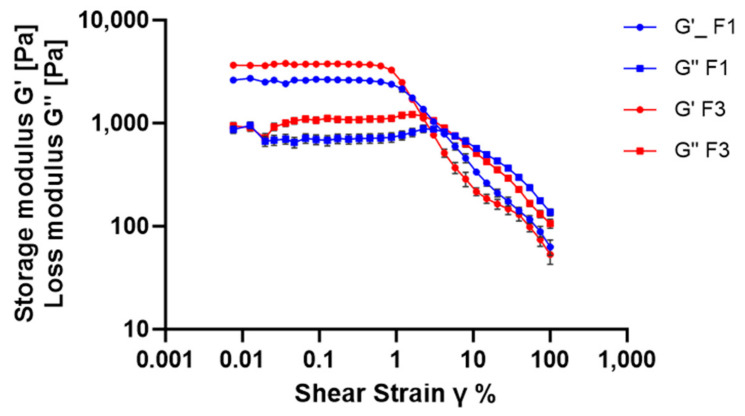
Amplitude sweep of F1 and F3: storage modulus (G′) and loss modulus (G″) as a function of strain (γ) at 10 rad/s. Rheological data are shown as mean ± SD (n = 3).

As shown in Figure 7, both F1 and F3 exhibited a decrease in G′ and an increase in G″ with increasing strain, indicating a transition to more fluid-like behavior under deformation, an important feature for spreadability on the skin [54,57]. The Linear Viscoelastic Region (LVR), where G′ remains constant, extended up to about 0.2% strain for both emulsions, with G′ values exceeding 10 Pa. These data confirm that both formulations possess well-structured internal networks, consistent with their classification as hard-base creams [52]. Additionally, the greater distance between the LVR limit and the G′ = G″ crossover point further reinforces the structural stability of both F1 and F3. The LVR behavior was not altered by the inclusion of RHS-H, indicating that the extract did not compromise the mechanical strength or structural integrity of the emulsion [54].

#### 3.5.3. Frequency Sweep Test

**Figure 8:**
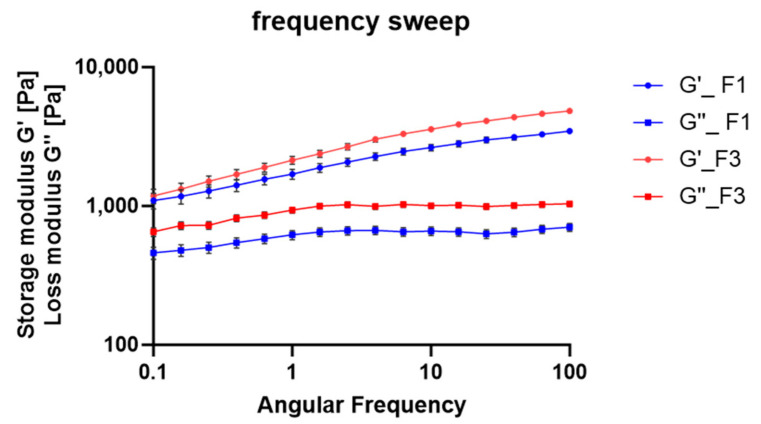
Frequency sweep of F1 (blue lines) and F3 (red lines): storage modulus (G′) and loss modulus (G″) plotted as a function of angular frequency (ω). Rheological data are shown as mean ± SD (n = 3).

The oscillatory frequency sweep tests revealed that both F1 and F3 exhibit predominantly solid-like behavior, as indicated by G′ consistently remaining higher than G″ across the applied frequency domain (Figure 8). The storage and loss moduli did not intersect at low angular frequencies, confirming the solid-like nature and stability of the emulsions by preventing particle or globule sedimentation and phase separation [57,58]. Emulsions are considered non-sticky when no crossover between the elastic and viscous moduli is observed, whereas a crossover indicates sticky behavior [58]. The high storage modulus (G′) values observed in both formulations are essential for ensuring structural stability over time. The linear viscoelastic properties of cosmetic emulsions are closely related to molecular interactions such as cross-linking, entanglement, and aggregation among components, and can be used to assess storage stability at rest. Cosmetic emulsions must maintain predominantly solid-like behavior to preserve their shape and integrity during shelf life. At an angular frequency of 0.1 rad/s, the G′/G″ ratio was 0.42 for F1 and 0.57 for F3, indicating that the elastic component dominates and reinforces their gel-like character. The storage modulus at low frequencies (ω ≤ 0.1 rad/s), referred to as G′_0_, provides further insight into the system’s stability at rest. Ideally, the G′ curve should show a negligible slope in this region. Experimental evidence suggests that for many dispersions and gels, G′_0_ ≥ 10 Pa (with G′ > G″) confirms a gel-like character and ensures stable dispersion. Conversely, G′_0_ ≤ 1 Pa indicates insufficient stability, while intermediate values require complementary tests such as yield stress and flow point analysis. In this study, the measured G′_0_ values were 995.23 Pa for F1 and 1083.2 Pa for F3, confirming excellent stability for both formulations, as their G′_0_ values largely exceeded the 10 Pa threshold. Additionally, the incorporation of RHS-H did not alter the intrinsic viscoelastic properties, as F3 maintained its elastic nature across the entire frequency range [59].

### 3.6. Evaluation of Antioxidant Activity of Emulsions F1–F4

Topical application of antioxidant-rich products is an effective strategy to counteract the depletion of endogenous antioxidants caused by environmental stressors such as pollution and UV exposure [60]. UV irradiation induces free radicals that impair skin barrier function, making antioxidants an essential component of sunscreen formulations. In addition to their direct protective role, antioxidants can enhance sunscreen efficacy by stabilizing UV filters, improving their photoprotection, and preventing oxidative degradation [35,61]. For example, Wu et al. 2011 [62] demonstrated that the combination of antioxidants, including ascorbyl phosphate, tocopherol acetate, *Echinacea pallida* extract, chamomile extract, and caffeine, with broad-spectrum sunscreens (benzophenone, butyl methoxydibenzoylmethane, avobenzone, and methoxycinnamate) significantly increased protection by reducing pigment formation, cytokeratin induction, UV-induced hyperproliferation, and the expression of photoaging markers such as matrix metalloproteinases (e.g., MMP-9).

**Figure 9:**
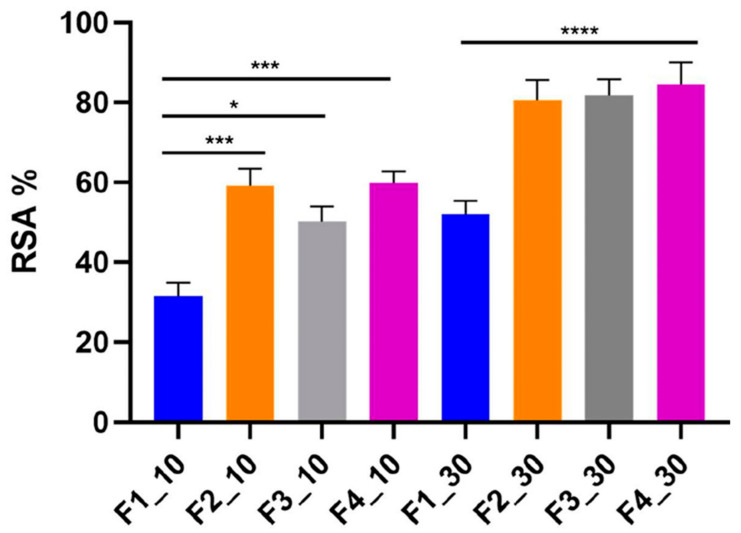
Radical Scavenging Activity (RSA, %) of formulations F1–F4 after 10 and 30 min of incubation with DPPH radical. Data are expressed as mean ± SD (n = 3). Data are expressed as mean ± SD of three independent experiments. * *p* < 0.05, *** *p* < 0.001, **** *p* < 0.0001 (one-way ANOVA with Tukey’s post hoc test).

In this study, the blank emulsion F1 exhibited radical scavenging activity (RSA) of 31.5% and 52.0% after 10 and 30 min of incubation with DPPH, respectively. This effect is likely due to ingredients with intrinsic antioxidant properties, such as *Persea gratissima* (avocado) oil and Butylated Hydroxytoluene (BHT). Avocado oil is naturally rich in tocopherols, carotenoids, and phytosterols, all of which can scavenge free radicals and protect skin lipids from peroxidation [63]. BHT, a synthetic phenolic antioxidant, is widely used in cosmetic and pharmaceutical products to stabilize lipids and prevent oxidative degradation [64]. The incorporation of RHS-H into emulsions F2, F3, and F4 significantly enhanced their radical scavenging capacity compared to F1. After 30 min of incubation, RSA increased from 52.0% in the blank formulation to 80.5%, 81.7%, and 84.5% in the RHS-H-enriched emulsions, respectively (Figure 9). These results confirm that RHS-H contributes substantially to the antioxidant potential of emulsions, complementing the activity of other formulation components and improving overall efficacy.

### 3.7. Assessment of RHS-H Preservative Performance in Developed O/W Emulsions: Challenge Test

Microbiological stability is a critical parameter to ensure the quality, safety, and shelf-life of cosmetic products. Contamination can alter the organoleptic and physicochemical characteristics of formulations, leading to degradation and potential adverse effects for consumers. To assess the preservative potential of RHS-H, emulsions F1 (synthetic preservative only, Phenoxyethanol + Ethylhexylglycerin, 1% *w*/*w*), F2 (RHS-H only, 0.2% *w*/*w*), and F3 (synthetic preservative 0.5% *w*/*w* + RHS-H 0.2% *w*/*w*) were subjected to a Challenge Test. The concentration of synthetic preservative in F3 was deliberately reduced below the manufacturer’s recommended range (0.6–1.0% *w*/*w*) to evaluate the potential co-preservative effect of RHS-H. The results were interpreted according to UNI EN ISO 11930:2022, which considers a formulation well preserved if it achieves: (i) a ≥3 log reduction in bacterial counts within 7 days (criteria A) or within 14 days (criteria B), (ii) a ≥1 log reduction in *Candida albicans* within 7 or 14 days, with no regrowth observed up to day 28, and (iii) no proliferation of *Aspergillus brasiliensis*, with a ≥1 log reduction required by day 28 (criteria A).

**Figure 10:**
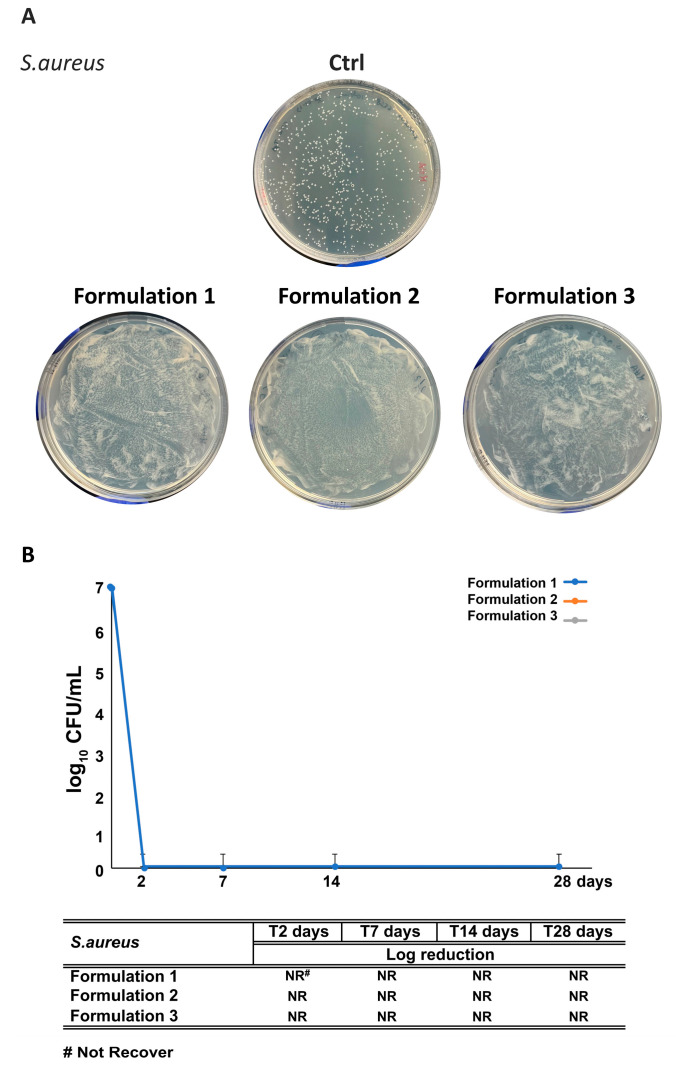
Challenge test with *S. aureus* of the formulations. (**A**) Representative images of plates following inoculation with *S. aureus* in F1 (synthetic preservative, 1% *w*/*w*), F2 (RHS-H, 0.2% *w*/*w*), and F3 (synthetic preservative, 0.5% *w*/*w* + RHS-H, 0.2% *w*/*w*). (**B**) Quantitative reduction of *S. aureus* at different time points (2, 7, 14, and 28 days) expressed as log_10_ CFU/mL. The table summarizes bacterial recovery, where “NR” indicates “No Recovery”. Each data point represents the mean ± standard deviation from triplicate experiments.

**Figure 11:**
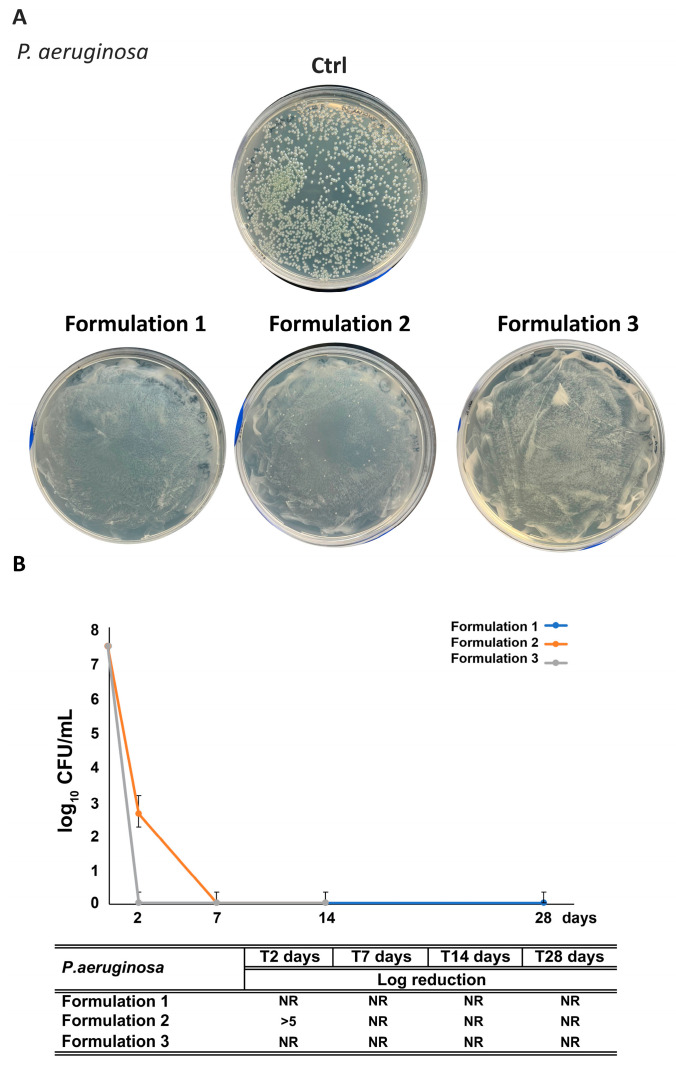
Challenge test with *P. aeruginosa* of the formulations. (**A**) Representative images of plates following inoculation with *P. aeruginosa*. (**B**) Quantitative reduction expressed as log_10_ CFU/mL for F1–F3 formulations at different time points (2, 7, 14, and 28 days). The table summarizes results, with “NR” = “No Recovery”. Each data point represents the mean ± standard deviation from triplicate experiments.

All formulations (F1–F3) satisfied criteria A for bacterial effectiveness. A complete inhibition of *Staphylococcus aureus* and *Pseudomonas aeruginosa* growth was observed in F1, F2, and F3 after 2 and 7 days, respectively (Figure 10 and Figure 11). Remarkably, RHS-H alone (F2) performed comparably to the synthetic preservative system (F1), confirming its intrinsic antibacterial activity. Notably, while raw RHS-H shows no effect against Gram-negative bacteria, its incorporation into the emulsion matrix enabled full protection against *P. aeruginosa*. This enhancement may result from formulation excipients: Nostro et al. 2002 [65] demonstrated that EDTA disrupts Gram-negative outer membranes by chelating Ca^2+^ and Mg^2+^ ions, thereby facilitating penetration of antimicrobial agents such as essential oils. A similar mechanism could explain the improved efficacy of RHS-H in emulsions.

**Figure 12:**
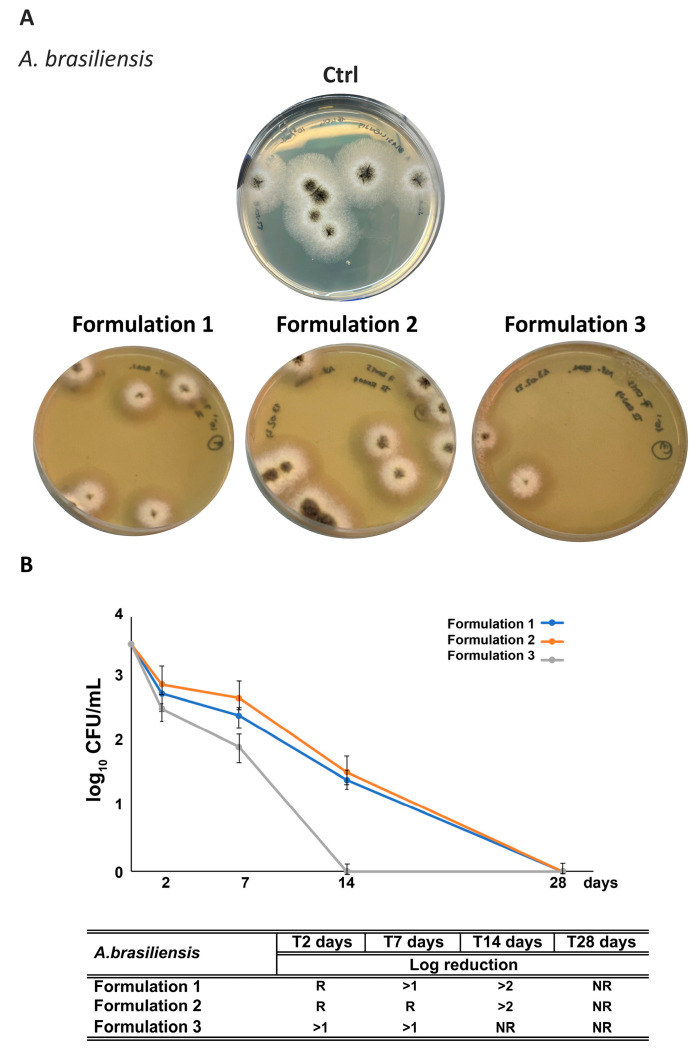
Challenge test with *A. brasiliensis* of the formulations. (**A**) Representative images of plates following inoculation with *A. brasiliensis*. (**B**) Quantitative reduction expressed as log_10_ CFU/mL for F1–F3 formulations at different time points (2, 7, 14, and 28 days). The table summarizes results, with “NR” indicates “No Recovery” of fungal growth, while “R” indicates “Recovery”. Each data point represents the mean ± standard deviation from triplicate experiments.

Regarding antifungal activity, all three formulations completely inhibited *A. brasiliensis* growth over 28 days (Figure 12). F3 achieved the most rapid response, reaching >1 log reduction by day 2, suggesting synergism between RHS-H and the reduced preservative dose. F2 alone displayed antifungal performance comparable to F1.

**Figure 13:**
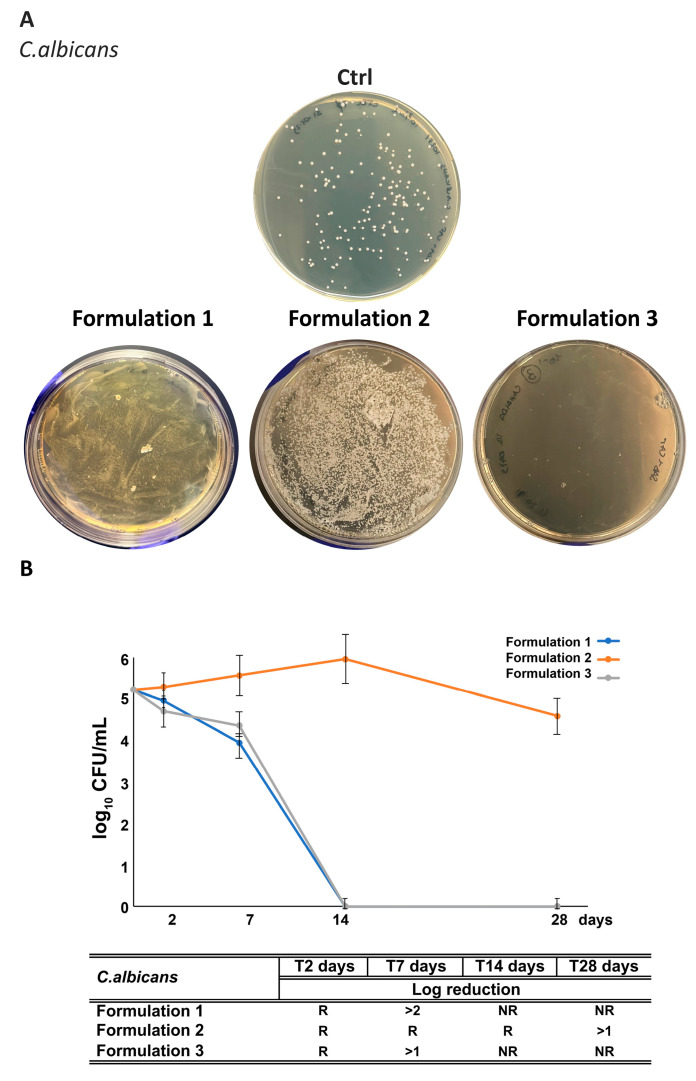
Challenge test with *C. albicans* of the formulations. (**A**) Representative images of plates following inoculation with *C. albicans*. (**B**) Quantitative reduction expressed as log_10_ CFU/mL for F1–F3 formulations at different time points (2, 7, 14, and 28 days). The table summarizes results, with “NR” indicates “No Recovery” of fungal growth, while “R” indicates “Recovery”. Each data point represents the mean ± standard deviation from triplicate experiments.

In contrast, the activity against *C. albicans* was more limited. RHS-H alone (F2) resulted in <1 log reduction after 14 days, insufficient to meet criterion A. However, F3 (RHS-H + half-dose preservative) achieved complete inhibition by day 14, satisfying criterion B (Figure 13). These results indicate that RHS-H enhances antifungal efficacy when combined with lower amounts of synthetic preservatives, acting as an effective co-preservative.

Taking together, these findings highlight that RHS-H provides strong preservative activity against *S. aureus*, *P. aeruginosa*, and *A. brasiliensis*, and acts as a co-preservative against *C. albicans*, enabling a reduction in synthetic preservative levels below the standard threshold required for efficacy. Importantly, product protection from *Candida* contamination could be further reinforced by adopting packaging strategies such as pump dispensers, pressurized containers, or single-dose units, which minimize exposure to air and microorganisms.

Our results are particularly promising given that other studies have reported preservative effects of plant extracts only at much higher concentrations (5–20% *w*/*w*). For example, Boukhira et al. 2017 [66] showed that *Silene vulgaris* extract (10–20% *w*/*w*) reduced microbial growth in creams, while Carvalho et al. 2024 [14] demonstrated preservative properties of sugarcane straw polyphenols in 5% emulsions. Similarly, *Morus nigra* leaf extracts (10–20% *w*/*w*) inhibited microbial growth within 7 days [67]. However, such high concentrations often compromise product aesthetics, leading to undesirable coloration (green to dark brown) and changes in viscosity or spreadability. By contrast, RHS-H proved effective at only 0.2% *w*/*w*, representing a significant advantage for the development of multifunctional phytocosmetics.

### 3.8. In Vitro Evaluation of SPF of O/W Cosmetic Formulations

Sun protection remains a major concern in dermatology and cosmetics due to the well-documented harmful effects of solar UV radiation. While UVC is absorbed by the ozone layer, both UVB and UVA contribute to erythema, photoaging, and skin cancer [68]. In this study, the UV protection efficacy of the developed formulations was assessed using the Diffey and Robson in vitro method, which provides a realistic approximation of SPF values by measuring product transmittance on a solid substrate.

**Figure 14:**
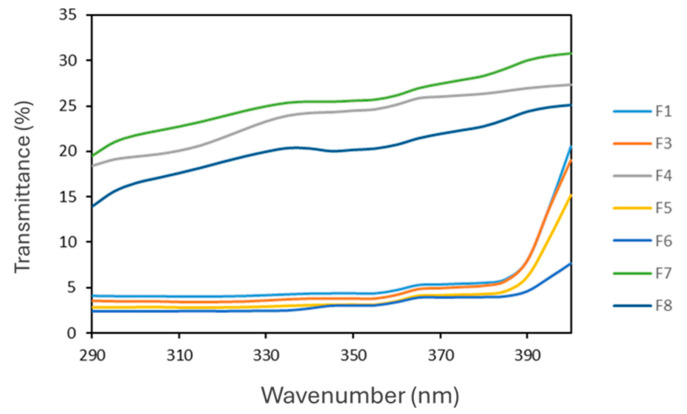
UV transmittance spectra of formulations F1, F3–F8 in the 290–400 nm range. Measurements were performed by Diffey and Robson in vitro method to assess SPF and broad-spectrum protection.

**Figure 15:**
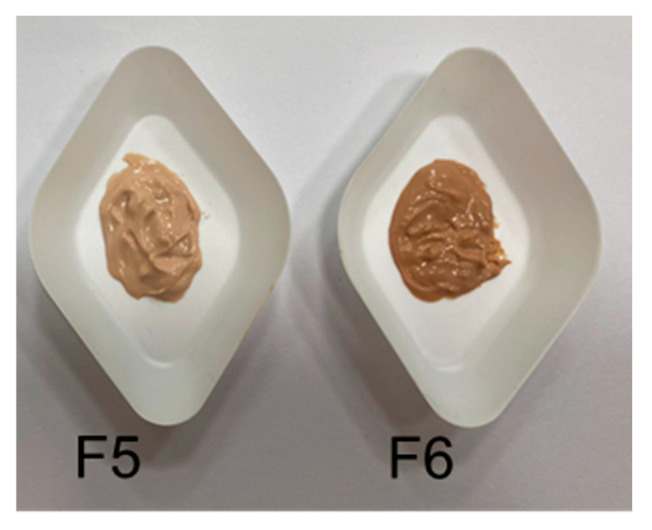
Macroscopic appearance of formulations containing 1% (F5) and 2% (F6) *w*/*w* RHS-H, showing the impact of extract concentration on the color of the cream.

No significant differences were observed in UV transmittance between F1 and F3 (Figure 14), both exhibiting high absorbance across the UVB and UVA ranges. The inclusion of RHS-H at 0.2% *w*/*w* produced a modest SPF increase, with F3 showing a 9.4% improvement over F1. More substantial enhancement was achieved at higher extract concentrations (1–2% *w*/*w* in F5 and F6), which resulted in SPF increases of 22.4% and 44.8%, respectively. However, these concentrations negatively affected the cream organoleptic properties, particularly their color (Figure 15).

Although RHS-H displayed UVB absorbance in solutions, within emulsions it acted as a broad-spectrum protector, extending coverage to both UVA and UVB regions (see F4 in Figure 14). This was confirmed by the critical wavelength values (>370 nm) and a UVA/UVB ratio close to 0.78 (Table 4).

The formulation F4, containing 0.2% RHS-H. would comply with the minimum requirement set by the U.S. FDA, which classifies a product as a sunscreen when the SPF is ≥ 2. In contrast, under the European regulation, sunscreens are defined as products with SPF values between 6 and 50 [69]. According to this higher standard, RHS-H alone did not provide sufficient photoprotective efficacy, as even at concentrations up to 2% *w*/*w* (F7 and F8), the SPF values remained below the threshold, reaching only 5.2 and 5.6, respectively.

These findings are consistent with previous studies on polyphenol-rich extracts. Hübner et al. 2020 [70] reported limited SPF for grape pomace extract used alone, but a significant SPF boost when combined with conventional UV filters. Similarly, Cefali et al. 2019 [54] observed that although flavonoid-rich extracts exhibited UVA and UVB absorbance in solutions, their incorporation into emulsions did not significantly enhance SPF [54]. This highlights a common limitation of natural phenolic compounds: despite their intrinsic UV-absorbing capacity, their SPF contribution in formulations is limited unless applied at high concentrations, which can compromise product aesthetics.

Nonetheless, natural extracts such as RHS-H remain of high interest because, beyond direct UV absorption, they may stabilize synthetic UV filters and provide additional cosmetic benefits, including antioxidant, antimicrobial, and anti-inflammatory activities [70,71].

## 4. Conclusions

Roasted hazelnut skins, traditionally regarded as an agro-industrial by-product, can be successfully upcycled into RHS-H, a natural multifunctional cosmetic ingredient. At low concentrations, RHS-H was successfully incorporated into O/W emulsions, enhancing antioxidant defense, photoprotection, and preservative performance. This sustainable extract offers a valuable opportunity to replace or complement synthetic additives, meeting industry needs for effective, green raw materials while aligning with consumer demand for safe, plant-based cosmetics.

## Figures and Tables

**Table 1:** Normalized values of the product function used in the Mansur Equation (1).

Wavelength (λ, nm)	EE × I Normalized
290	0.0150
295	0.0817
300	0.2874
305	0.3278
310	0.1864
315	0.0839
320	0.0180
Total	1

**Table 2:** Qualitative and quantitative of O/W emulsions F1–F4. F1: control with synthetic UV filters and preservatives; F2: RHS-H as preservative; F3: RHS-H as co-preservative with synthetic filters/preservatives; F4: RHS-H as UV-protective agent.

Ingredient (INCI Name)	Function	F1	F2	F3	F4
		Concentration % (*w*/*w*)			
Aqueous phase (A)					
Aqua	Solvent	*qs. 100	qs. 100	qs. 100	qs. 100
Glycerin	Humectant	4.00	3.00	3.00	3.00
Xanthan Gum	Thickening agent	0.25	0.25	0.25	0.25
Disodium EDTA	Chelating agent	0.10	0.10	0.10	0.10
Oil phase (B)					
Butyrospermum parkii	Skin conditioning/ Consistency regulator	3.00	3.00	3.00	4.00
Glyceryl Stearate (and) Peg-100 Stearate ^1^	Emulsifier	5.00	5.00	5.00	5.00
Cetearyl Alcohol	Emulsion stabilising	3.00	3.00	3.00	3.00
Ethylhexyl Stearate	Emollient	2.00	2.00	2.00	2.00
Persea Gratissima Oil	Skin conditioning	1.00	1.00	1.00	3.00
Dimethicone	Antifoaming	0.50	0.50	0.50	0.50
Caprylic/Capric Triglyceride	Skin conditioning	3.00	3.00	3.00	3.00
Dicaprylyl Ether	Emollient	1.00	1.00	1.00	1.00
C12-15 Alkyl Benzoate	Solubilizer for UV filters/Emollient	8.00	8.00	8.00	8.00
Ethylhexyl Methoxycinnamate	UV-B filter	8.00	8.00	8.00	-
Butyl Methoxydibenzoylmethane	UV-A filter	4.70	4.70	4.70	-
Octocrylene Butylhydroxytoluene	UV-B filter Antioxidant	8.00 0.01	8.00 0.01	8.00 0.01	- 0.01
Phase C					
Phenoxyethanol (and) Ethylhexylglycerin	Synthetic Preservative	1.00	-	0.50	1.00
Phase D					
RHS-H	Roasted Hazelnut skin hydroalcoholic extract	-	0.20	0.20	0.20
Glycerin	Humectant	-	1.00	1.00	1.00
Aqua	Solvent	-	1.00	1.00	1.00

*qs. = quantum sufficiency; ^1^ The breakdown of this ingredient is Glyceryl Stearate (50%), PEG-100 Stearate (50%).

**Table 3:** In vitro Sun Protection Factor (SPF) values of RHS-H and its combinations with synthetic UV filters. SPF values were calculated using the Mansur equation. RHS-H enhanced the SPF of all tested filters when used as a booster at 0.1 mg/mL.

Sunscreen Ingredient (Concentration)	UV-Filter Booster	In Vitro SPF (Mansur Equation)
RHS-H (0.1 mg/mL)	-	3.63 ± 0.11
Octocrylene (0.01 mg/mL)	-	4.90 ± 0.12
Ethylhexyl Methoxycinnamate (0.005 mg/mL)	-	3.88 ± 0.49
Butyl Methoxydibenzoylmethane (0.01 mg/mL)	-	3.98 ± 0.11
Octocrylene (0.01 mg/mL)	RHS-H (0.1 mg/mL)	9.13 ± 0.91
Ethylhexyl Methoxycinnamate (0.005 mg/mL)	RHS-H (0.1 mg/mL)	9.73 ± 0.57
Butyl Methoxydibenzoylmethane (0.01 mg/mL)	RHS-H (0.1 mg/mL)	7.33 ± 0.58

**Table 4:** In vitro photoprotection parameters of O/W emulsions containing RHS-H: Sun Protection Factor (SPF), UVA/UVB ratio, and critical wavelength (C_λ_). Values are reported as mean ± standard deviation (n = 3).

Emulsion System	SPF	UVA/UVB	C_λ_
F1	27.7 ± 1.2	0.82	385
F3	30.3 ± 1.7	0.81	385
F4	5.2 ± 1.3	0.78	388
F5	33.9 ± 1.2	0.81	397
F6	40.1 ± 2.3	0.83	395
F7	4.3 ± 0.5	0.79	391
F8	5.6 ± 0.2	0.78	391

## Data Availability

Data are contained within the article.

## Abbreviations

The following abbreviations are used in this manuscript:

RHS	Roasted Hazelnut Skins
RHS-H	Roasted Hazelnut Skin Hydroalcoholic extract
GAE	Gallic Acid Equivalents
CE	Catechin Equivalents
DPPH	1,1-Diphenyl-2-picrylhydrazyl radical
TEAC	Trolox Equivalent Antioxidant Capacity
UV	Ultraviolet
O/W	Oil in Water
SPF	Sun Protection Factor
ROS	Reactive Oxygen Species
DNA	Deoxyribonucleic acid
PBS	Phosphate-Buffered Saline
ABTS	2,2′-Azino-bis(3-ethylbenzothiazoline-6-sulfonic acid)
EDTA	Ethylenediaminetetraacetic acid
PLE	Pressurized Liquid Extraction
HPLC-UV-HRMS	High Performance Liquid Chromatography-Ultraviolet-High Resolution Mass Spectrometry
HPLC-UV	High Performance Liquid Chromatography-Ultraviolet
EC50	Effective scavenger Concentration50
TE	Trolox Equivalents
MBC	Minimum Bactericidal Concentration
MHB	Mueller–Hinton Broth
MHA	Mueller–Hinton Agar
ELS	Electrophoretic Light Scattering
LVR	Linear Viscoelastic Region
RSA	Radical Scavenging Activity
PAs	Proanthocyanidins
mDP	average Degree of Polymerization
CTRL	untreated control
EtOH	Ethanol
BHT	Butylated Hydroxytoluene
NR	No Recovery
R	Recovery
FDA	Food and Drug Administration

## References

[1] Piccinelli A.L., Pagano I., Esposito T., Mencherini T., Porta A., Petrone A.M., Gazzerro P., Picerno P., Sansone F., Rastrelli L. (2016). HRMS Profile of a Hazelnut Skin Proanthocyanidin-Rich Fraction with Antioxidant and Anti-Candida Albicans Activities. J. Agric. Food Chem..

[2] Spagnuolo L., Della Posta S., Fanali C., Dugo L., De Gara L., Gugliucci A. (2021). Antioxidant and Antiglycation Effects of Polyphenol Compounds Extracted from Hazelnut Skin on Advanced Glycation End-Products (AGEs) Formation. Antioxidants.

[3] Zhao J., Wang X., Lin H., Lin Z. (2023). Hazelnut and Its By-Products: A Comprehensive Review of Nutrition, Phytochemical Profile, Extraction, Bioactivities and Applications. Food Chem..

[4] Frazzini S., Zuorro A., Panseri S., Pavlovic R., Sgoifo Rossi C.A., Rossi L. (2023). Repurposing Hazelnut Waste Products for a Sustainable Economy: A Metabolomic Analysis of Cuticles and Shells to Highlight Their Antioxidant Potential and Inhibitory Activity against Verocytotoxic *Escherichia coli*. Sustainability.

[5] Nie F., Liu L., Cui J., Zhao Y., Zhang D., Zhou D., Wu J., Li B., Wang T., Li M. (2023). Oligomeric Proanthocyanidins: An Updated Review of Their Natural Sources, Synthesis, and Potentials. Antioxidants.

[6] (2009). Regulation (EC) No 1223/2009 of the European Parliament and of the Council on Cosmetic Products.

[7] Rathee P., Sehrawat R., Rathee P., Khatkar A., Akkol E.K., Khatkar S., Redhu N., Türkcanoğlu G., Sobarzo-Sánchez E. (2023). Polyphenols: Natural Preservatives with Promising Applications in Food, Cosmetics and Pharma Industries; Problems and Toxicity Associated with Synthetic Preservatives; Impact of Misleading Advertisements; Recent Trends in Preservation and Legislation. Materials.

[8] Jesus A., Sousa E., Cruz M.T., Cidade H., Lobo J.M.S., Almeida I.F. (2022). UV Filters: Challenges and Prospects. Pharmaceuticals.

[9] Pourzand C., Albieri-Borges A., Raczek N.N. (2022). Shedding a New Light on Skin Aging, Iron-and Redox-Homeostasis and Emerging Natural Antioxidants. Antioxidants.

[10] Masaki H. (2010). Role of Antioxidants in the Skin: Anti-Aging Effects. J. Dermatol. Sci..

[11] Petruk G., Del Giudice R., Rigano M.M., Monti D.M. (2018). Antioxidants from Plants Protect against Skin Photoaging. Oxidative Med. Cell. Longev..

[12] Ngoc L.T.N., van Tran V., Moon J.Y., Chae M., Park D., Lee Y.C. (2019). Recent Trends of Sunscreen Cosmetic: An Update Review. Cosmetics.

[13] Saewan N., Jimtaisong A. (2015). Natural Products as Photoprotection. J. Cosmet. Dermatol..

[14] Carvalho M.J., Pedrosa S.S., Pintado M., Oliveira A.L.S., Madureira A.R. (2024). New Natural and Sustainable Cosmetic Preservative Based on Sugarcane Straw Extract. Molecules.

[15] Silvério L.A.L., Coco J.C., de Macedo L.M., dos Santos É.M., Sueiro A.C., Ataide J.A., Tavares G.D., Paiva-Santos A.C., Mazzola P.G. (2023). Natural Product-Based Excipients for Topical Green Formulations. Sustain. Chem. Pharm..

[16] Zagoskina N.V., Zubova M.Y., Nechaeva T.L., Kazantseva V.V., Goncharuk E.A., Katanskaya V.M., Baranova E.N., Aksenova M.A. (2023). Polyphenols in Plants: Structure, Biosynthesis, Abiotic Stress Regulation, and Practical Applications (Review). Int. J. Mol. Sci..

[17] Gomez-Molina M., Albaladejo-Marico L., Yepes-Molina L., Nicolas-Espinosa J., Navarro-León E., Garcia-Ibañez P., Carvajal M. (2024). Exploring Phenolic Compounds in Crop By-Products for Cosmetic Efficacy. Int. J. Mol. Sci..

[18] Matos M.S., Romero-Díez R., Álvarez A., Bronze M.R., Rodríguez-Rojo S., Mato R.B., Cocero M.J., Matias A.A. (2019). Polyphenol-Rich Extracts Obtained from Winemakingwaste Streams as Natural Ingredients with Cosmeceutical Potential. Antioxidants.

[19] Farhan M. (2024). The Promising Role of Polyphenols in Skin Disorders. Molecules.

[20] Gǎlbǎu C.Ş., Irimie M., Neculau A.E., Dima L., Pogačnik da Silva L., Vârciu M., Badea M. (2024). The Potential of Plant Extracts Used in Cosmetic Product Applications—Antioxidants Delivery and Mechanism of Actions. Antioxidants.

[21] Esposito T., Silva N.H.C.S., Almeida A., Silvestre A.J.D., Piccinelli A., Aquino R.P., Sansone F., Mencherini T., Vilela C., Freire C.S.R. (2020). Valorisation of Chestnut Spiny Burs and Roasted Hazelnut Skins Extracts as Bioactive Additives for Packaging Films. Ind. Crops Prod..

[22] Butler L.G., Price M.L., Brotherton J.E. (1982). Vanillin Assay for Proanthocyanidins (Condensed Tannins): Modification of the Solvent for Estimation of the Degree of Polymerization. J. Agric. Food Chem..

[23] Gu L., Kelm M., Hammerstone J.F., Beecher G., Cunningham D., Vannozzi S., Prior R.L. (2002). Fractionation of Polymeric Procyanidins from Lowbush Blueberry and Quantification of Procyanidins in Selected Foods with an Optimized Normal-Phase HPLC-MS Fluorescent Detection Method. J. Agric. Food Chem..

[24] Esposito T., Celano R., Pane C., Piccinelli A.L., Sansone F., Picerno P., Zaccardelli M., Aquino R.P., Mencherini T. (2019). Chestnut (*Castanea sativa* Miller.) Burs Extracts and Functional Compounds: Uhplc-Uv-Hrms Profiling, Antioxidant Activity, and Inhibitory Effects on Phytopathogenic Fungi. Molecules.

[25] Reis Mansur M.C.P.P., Leitão S.G., Cerqueira-Coutinho C., Vermelho A.B., Silva R.S., Presgrave O.A.F., Leitão Á.A.C., Leitão G.G., Ricci-Júnior E., Santos E.P. (2016). In Vitro and in Vivo Evaluation of Efficacy and Safety of Photoprotective Formulations Containing Antioxidant Extracts. Rev. Bras. Farmacogn..

[26] Sayre R.M., Agin P.P., Levee G.J., Maruiwe E. (1979). A comparison of in vivo and in vitro testing of sunscreening formulas. Photochem. Photobiol..

[27] Esposito T., Mencherini T., Sansone F., Auriemma G., Gazzerro P., Puca R.V., Iandoli R., Aquino R.P. (2021). Development, Characterization, and Clinical Investigation of a New Topical Emulsion System Containing a *Castanea Sativa* Spiny Burs Active Extract. Pharmaceutics.

[28] Serra M., Botelho C., Almeida H., Casas A., Teixeira J.A., Barros A.N. (2025). Stable and Functional Cosmetic Creams Enriched with Grape Stem Extract: A Sustainable Skincare Strategy. Antioxidants.

[29] Caruso C., Porta A., Tosco A., Eletto D., Pacente L., Bartollino S., Costagliola C. (2020). A Novel Vitamin E TPGS-Based Formulation Enhances Chlorhexidine Bioavailability in Corneal Layers. Pharmaceutics.

[30] Diffey B.L., Robson J. (1989). A New Substrate to Measure Sunscreen Protection Factors throughout the Ultraviolet Spectrum. J. Soc. Cosmet. Chem..

[31] Michalak M. (2022). Plant-Derived Antioxidants: Significance in Skin Health and the Ageing Process. Int. J. Mol. Sci..

[32] Bencresciuto G.F., Carnevale M., Paris E., Gallucci F., Santangelo E., Migliori C.A. (2025). A Sustainable Alternative for Cosmetic Applications: NADES Extraction of Bioactive Compounds from Hazelnut By-Products. Sustainability.

[33] Ku C.S., Mun S.P. (2007). Characterization of Proanthocyanidin in Hot Water Extract Isolated from Pinus Radiata Bark. Wood Sci. Technol..

[34] Ivanović S., Avramović N., Dojčinović B., Trifunović S., Novaković M., Tešević V., Mandić B. (2020). Chemical Composition, Total Phenols and Flavonoids Contents and Antioxidant Activity as Nutritive Potential of Roasted Hazelnut Skins (*Corylus avellana* L.). Foods.

[35] Galanakis C.M., Tsatalas P., Galanakis I.M. (2018). Phenols from Olive Mill Wastewater and Other Natural Antioxidants as UV Filters in Sunscreens. Environ. Technol. Innov..

[36] Galanakis C.M., Tsatalas P., Galanakis I.M. (2018). Implementation of Phenols Recovered from Olive Mill Wastewater as UV Booster in Cosmetics. Ind. Crops Prod..

[37] Gaweł-Bęben K., Kukula-Koch W., Hoian U., Czop M., Strzępek-Gomółka M., Antosiewicz B. (2020). Characterization of *Cistus × incanus* L. and *Cistus ladanifer* L. Extracts as Potential Multifunctional Antioxidant Ingredients for Skin Protecting Cosmetics. Antioxidants.

[38] Liu Z., Liao H., Dai Y., Qi Y., Zou Z. (2024). Characterization and Anti-Ultraviolet Radiation Activity of Proanthocyanidin-Rich Extracts from *Cinnamomum camphora* by Ultrasonic-Assisted Method. Molecules.

[39] Ferreira S.M., Gomes S.M., Santos L. (2023). A Novel Approach in Skin Care: By-Product Extracts as Natural UV Filters and an Alternative to Synthetic Ones. Molecules.

[40] Era B., Floris S., Sogos V., Porcedda C., Piras A., Medda R., Fais A., Pintus F. (2021). Anti-Aging Potential of Extracts from *Washingtonia filifera* Seeds. Plants.

[41] Krishna S., Miller L.S. (2012). Host-Pathogen Interactions between the Skin and Staphylococcus Aureus. Curr. Opin. Microbiol..

[42] Severn M.M., Horswill A.R. (2023). Staphylococcus Epidermidis and Its Dual Lifestyle in Skin Health and Infection. Nat. Rev. Microbiol..

[43] Di Michele A., Pagano C., Allegrini A., Blasi F., Cossignani L., Di Raimo E., Faieta M., Oliva E., Pittia P., Primavilla S. (2021). Hazelnut Shells as Source of Active Ingredients: Extracts Preparation and Characterization. Molecules.

[44] Harfoush A., Swaidan A., Khazaal S., Salem Sokhn E., Grimi N., Debs E., Louka N., El Darra N. (2024). From Spent Black and Green Tea to Potential Health Boosters: Optimization of Polyphenol Extraction and Assessment of Their Antioxidant and Antibacterial Activities. Antioxidants.

[45] Nitulescu G., Lupuliasa D., Adam-Dima I., Nitulescu G.M. (2023). Ultraviolet Filters for Cosmetic Applications. Cosmetics.

[46] Pinto D., Lameirão F., Delerue-Matos C., Rodrigues F., Costa P. (2021). Characterization and Stability of a Formulation Containing Antioxidants-Enriched *Castanea sativa* Shells Extract. Cosmetics.

[47] Tang Z., Du Q. (2024). Mechanism of Action of Preservatives in Cosmetics. J. Dermatol. Sci. Cosmet. Technol..

[48] Singh S., Lohani A., Mishra A.K., Verma A. (2019). Formulation and Evaluation of Carrot Seed Oil-Based Cosmetic Emulsions. J. Cosmet. Laser Ther..

[49] Mirhosseini H., Tan C.P., Hamid N.S.A., Yusof S. (2008). Effect of Arabic Gum, Xanthan Gum and Orange Oil Contents on ζ-Potential, Conductivity, Stability, Size Index and PH of Orange Beverage Emulsion. Colloids Surf. A Physicochem. Eng. Asp..

[50] Saharudin S.H., Ahmad Z. (2016). Role of Xanthan Gum on Physicochemical and Rheological Properties of Rice Bran Oil Emulsion. Int. Food Res. J..

[51] de Oliveira Paulo L.A., Fernandes R.N., Simiqueli A.A., Rocha F., dos Santos Dias M.M., Minim V.P.R., Minim L.A., Vidigal M.C.T.R. (2023). Baru Oil (*Dipteryx alata* Vog.) Applied in the Formation of O/W Nanoemulsions: A Study of Physical-Chemical, Rheological and Interfacial Properties. Food Res. Int..

[52] Danila A., Ibanescu S.A., Zaharia C., Muresan E.I., Popescu A., Danu M., Rotaru V. (2021). Eco-Friendly O/W Emulsions with Potential Application in Skincare Products. Colloids Surf. A Physicochem. Eng. Asp..

[53] Huynh A., Garcia A.G., Young L.K., Szoboszlai M., Liberatore M.W., Baki G. (2021). Measurements Meet Perceptions: Rheology–Texture–Sensory Relations When Using Green, Bio-Derived Emollients in Cosmetic Emulsions. Int. J. Cosmet. Sci..

[54] Cefali L.C., Ataide J.A., Fernandes A.R., Sousa I.M.D.O., Gonçalves F.C.D.S., Eberlin S., Dávila J.L., Jozala A.F., Chaud M.V., Sanchez-Lopez E. (2019). Flavonoid-Enriched Plant-Extract-Loaded Emulsion: A Novel Phytocosmetic Sunscreen Formulation with Antioxidant Properties. Antioxidants.

[55] López-Hortas L., Falqué E., Domínguez H., Torres M.D. (2020). Microwave Hydrodiffusion and Gravity versus Conventional Distillation for Acacia Dealbata Flowers. Recovery of Bioactive Extracts for Cosmetic Purposes. J. Clean. Prod..

[56] Ibănescu C., Danu M., Nanu A., Lungu M., Simionescu B.C. (2010). Stability of Disperse Systems Estimated Using Rheological Oscillatory Shear Tests. Rev. Roum. Chim.

[57] Turcov D., Barna A.S., Blaga A.C., Ibanescu C., Danu M., Trifan A., Zbranca A., Suteu D. (2022). Dermatocosmetic Emulsions Based on Resveratrol, Ferulic Acid and Saffron (*Crocus sativus*) Extract to Combat Skin Oxidative Stress-Trigger Factor of Some Potential Malignant Effects: Stability Studies and Rheological Properties. Pharmaceutics.

[58] Adejokun D.A., Dodou K. (2020). Quantitative Sensory Interpretation of Rheological Parameters of a Cream Formulation. Cosmetics.

[59] Mieles-Gómez L., Lastra-Ripoll S.E., Torregroza-Fuentes E., Quintana S.E., García-Zapateiro L.A. (2021). Rheological and Microstructural Properties of Oil-in-Water Emulsion Gels Containing Natural Plant Extracts Stabilized with Carboxymethyl Cellulose/Mango (*Mangifera indica*) Starch. Fluids.

[60] Irato P., Santovito G. (2021). Enzymatic and Non-Enzymatic Molecules with Antioxidant Function. Antioxidants.

[61] Vilela F.M.P., Oliveira F.M., Vicentini F.T.M.C., Casagrande R., Verri W.A., Cunha T.M., Fonseca M.J.V. (2016). Commercial Sunscreen Formulations: UVB Irradiation Stability and Effect on UVB Irradiation-Induced Skin Oxidative Stress and Inflammation. J. Photochem. Photobiol. B.

[62] Wu Y., Matsui M.S., Chen J.Z.S., Jin X., Shu C.M., Jin G.Y., Dong G.H., Wang Y.K., Gao X.H., Chen H.D. (2011). Antioxidants Add Protection to a Broad-Spectrum Sunscreen. Clin. Exp. Dermatol..

[63] Ferreira S.M., Falé Z., Santos L. (2022). Sustainability in Skin Care: Incorporation of Avocado Peel Extracts in Topical Formulations. Molecules.

[64] Karioti A., Furlan C., Vincieri F.F., Bilia A.R. (2011). Analysis of the Constituents and Quality Control of Viola Odorata Aqueous Preparations by HPLC-DAD and HPLC-ESI-MS. Anal. Bioanal. Chem..

[65] Nostro A., Cannatelli M.A., Morelli I., Cioni P.L., Bader A., Marino A., Alonzo V. (2002). Preservative Properties of Calamintha Officinalis Essential Oil with and without EDTA. Lett. Appl. Microbiol..

[66] Boukhira S., Balouiri M., El Mansouri L., El Youbi A.E.H., Bouarfa M., Lebtar S., Ouhammou A., Bousta D. (2017). Development of Natural Preservative from Silene Vulgaris Extract in Topical Formulation under a Challenge Test and Its Stability Study. J. Appl. Pharm. Sci..

[67] de Melo R.S., Reis S.A.G.B., Guimarães A.L., Silva N.D.D.S., Rocha J.M., El Aouad N., Almeida J.R.G. (2022). da S. Phytocosmetic Emulsion Containing Extract of *Morus nigra* L. (Moraceae): Development, Stability Study, Antioxidant and Antibacterial Activities. Cosmetics.

[68] Verma A., Zanoletti A., Kareem K.Y., Adelodun B., Kumar P., Ajibade F.O., Silva L.F.O., Phillips A.J., Kartheeswaran T., Bontempi E. (2024). Skin Protection from Solar Ultraviolet Radiation Using Natural Compounds: A Review. Environ. Chem. Lett..

[69] Couteau C., Coiffard L. (2016). About Suncare Products. Chemistry Research Summaries.

[70] Hübner A.A., Sarruf F.D., Oliveira C.A., Neto A.V., Fischer D.C.H., Kato E.T.M., Lourenço F.R., Baby A.R., Bacchi E.M. (2020). Safety and Photoprotective Efficacy of a Sunscreen System Based on Grape Pomace (*Vitis vinifera* L.) Phenolics from Winemaking. Pharmaceutics.

[71] Morocho-Jácome A.L., Freire T.B., de Oliveira A.C., de Almeida T.S., Rosado C., Velasco M.V.R., Baby A.R. (2021). In Vivo SPF from Multifunctional Sunscreen Systems Developed with Natural Compounds—A Review. J. Cosmet. Dermatol..

